# Studies with neutralizing antibodies suggest CXCL8-mediated neutrophil activation is independent of C-C motif chemokine receptor-like 2 (CCRL2) ligand binding function

**DOI:** 10.1371/journal.pone.0280590

**Published:** 2023-01-20

**Authors:** Zhenwei Su, Jonathan Brooks, Jeffrey Pelker, Tatyana Andreyeva, Hanna Sobon, Roger Gifford, Matthew Powers, Jing Wang, Corey Dower, Martin Hegen, Dean Messing, Alfredo Darmanin Sheehan, Joseph J. Brennan

**Affiliations:** 1 BioMedicine Design, Pfizer, Cambridge, Massachusetts, United States of America; 2 Inflammation and Immunology, Pfizer, Cambridge, Massachusetts, United States of America; 3 BioMedicine Design, Pfizer, Dublin, Ireland; Lewis Katz School of Medicine, Temple University, UNITED STATES

## Abstract

C-C motif chemokine receptor-like 2 (CCRL2) is a non-signaling 7 transmembrane receptor that binds chemotactic ligands to shape leukocyte recruitment to sites of inflammation. However, there is a lack of consensus on the ligands that directly bind CCRL2 or their functional impact. Studies with CCRL2 knockout mice have demonstrated that neutrophils have impaired degranulation and migration in response to CXCL8, where the underlying molecular mechanism is proposed to be due to the formation of CCRL2 heterodimers with the chemokine receptor CXCR2. Herein, we characterized the ligands that bind directly to CCRL2 and interrogated the impact of CCRL2 neutralization on CXCL8 signaling in neutrophils using pharmacological antibody tools. Using flow cytometry and Surface Plasmon Resonance microscopy (SPRm) cell binding experiments, we confirmed that chemerin, but not previously reported C-C chemokines, binds CCRL2. Furthermore, we identified human and mouse CCRL2 antibodies that neutralized chemerin binding to CCRL2. Unexpectedly, we found that neutralization of CCRL2 with these antibodies did not attenuate CXCL8-induced human neutrophil degranulation nor CXCL8-induced murine neutrophil recruitment to the peritoneum. Based on the observed differences in modulating CCRL2 function with neutralizing antibodies compared to the reported CCRL2 deficient murine models, we hypothesize that the ligand binding function of CCRL2 is dispensable for CXCL8 signaling in neutrophils. Finally, extensive profiling of CCRL2 expression on peripheral blood leukocytes revealed monocytes, dendritic cells (DC), and subpopulations of natural killer T (NKT) cells as additional targets, highlighting potential roles for CCRL2 in human cell types beyond neutrophils that warrants future investigation.

## Introduction

Chemoattractant receptors and their cognate ligands shape the localization and recruitment of leukocytes in vivo and have instrumental roles for promoting and resolving inflammation. Most chemoattractant receptors are conventional seven transmembrane-spanning (7TM) G-coupled protein receptors (GPCRs) that, upon binding ligands, initiate intracellular signaling to direct leukocyte migration and localization [1, 2]. However, these conventional GPCRs are not the only chemoattractant receptors capable of binding chemokines. Atypical chemokine receptors (ACKRs) have a high degree of homology to conventional chemokine receptors and bind chemokines. Unlike conventional GPCRs, ACKRs do not activate G-protein dependent signaling required to direct cell migration [3, 4]. Rather, ACKRs modulate chemokine availability by acting as scavenger receptors, promoting chemokine endocytosis, or creating chemokine gradients [4–6]. As such, ACKRs have been studied for their roles in regulating cell migration during inflammation, as well as in tumor biology and tumor evasion from host immune defenses [5, 7]. Currently, the ACKR family includes 4 members, ACKR1 (Duffy antigen receptor for chemokines), ACKR2 (D6 or BP2), ACKR3 (CXCR7 or RDC1) and ACKR4 (CCRL1 or CCXCKR) [5].

C-C motif chemokine receptor-like 2 (CCRL2) is a non-signaling 7TM receptor related to the ACKR family. However, CCRL2 differs from ACKRs because it is devoid of ligand scavenger functions and lacks confirmed binding to classical chemokines [8]. Several chemotactic ligands have been proposed for CCRL2 including the non-chemokine chemotactic protein chemerin, and the C-C chemokines CCL2, CCL5, CCL7, CCL8, CCL18, CCL19, CCL21 [9–12]. Among these ligands, chemerin has been the most widely studied as a direct CCRL2 ligand [9–13]. CCRL2 is expressed in endothelial cells, epithelial cells and several leukocytes including mast cells, neutrophils and dendritic cells (DCs) [8], however, the function of CCRL2 remains largely elusive. To date, two molecular mechanisms have been proposed for CCRL2 in regulating leukocyte migration. First, endothelial or epithelial cell expressed CCRL2 binds and presents chemerin to Chemerin Chemokine-like Receptor 1 (CMKLR1), which can direct natural killer (NK) cell and DC recruitment and transmigration [8, 14–16]. Second, CCRL2 forms heterodimers with CXCR2 to promote neutrophil activation and recruitment in response to CXCL8 [8, 17]. However, the interrogation of these mechanisms has largely relied on the utilization of recombinant expression systems or CCRL2 deficient mice [8, 16, 17]. As such, there is a need to identify pharmacological tools to further study the roles of CCRL2 in human cells and in vivo.

Recent technological advancements have facilitated the characterization of ligand-receptor interactions and the identification of neutralizing antibodies to interrogate the function of cell surface receptors under native expression conditions. Flow cytometry has been a long-standing method to enable high-throughput characterization of receptor-ligand pairs and antibody binding to cell surface receptors [18, 19]. A recently developed platform, Surface Plasmon Resonance Microscopy (SPRm), measures the kinetics of molecular interactions to the surface of mammalian cells and has been crucial for determining on-rate (k_on_), off-rate (k_off_) and binding affinity (K_d_) for antibodies to cell-surface expressed targets [20–22]. Since SPRm does not require tagged or labeled analytes, it is beneficial for mitigating potential effects of labeling on analyte function and affinity.

In this study, we sought to ascertain ligands that directly bind CCRL2 and identify tool antibodies that neutralize CCRL2 function based on their ability to inhibit ligand binding to CCRL2. We then explored the impacts of these antibodies on neutrophil responses to CXCL8 in vitro and in vivo. Additionally, our analyses of CCRL2 expression among leukocytes revealed distinct cell populations that express CCRL2, highlighting unexplored roles for CCRL2 to direct cellular migration and activation.

## Materials and methods

### Human and murine CCRL2 overexpression cell line generation

Codon optimized human (hu) and mouse (mu) CCRL2 isoform b (Genewiz, South Plainfield, NJ) were cloned into the retroviral expression vector pQCXIN (Clontech Laboratories Inc., Mountain View, CA) and co-transfected with Amphotropic Envelope Vector (Cell Biolabs Inc., San Diego, CA) into HEK 293T cells (ATCC, Manassas, VA; CRL-3216). Positive clones were selected using 1 mg/ml G418 (ThermoFisher Scientific, Waltham, MA).

### Ligand and antibody information

To generate recombinant human and murine chemerin, hIgG1 monovalent Fc-TEV-chemerin fusion constructs were subcloned using active human chemerin (Uniprot entry Q99969; residues 21–157) and active mouse chemerin (Uniprot entry Q9DD06; residues 21–156). Constructs were transiently expressed in HEK 293F cells (ThermoFisher Scientific) at 28°C, 5% CO_2_. Cultures were centrifuged for 30 min at 3000 rpm, clarified by 0.2 μm filtration and monovalent Fc-TEV-chemerin fusions were affinity purified using MabSelect SuRe Protein A resin (Cytiva, Marlborough, MA). Protein A eluates were buffer exchanged (20 mM Tris, 150 mM NaCl, pH 8.0) and Fc-TEV-chemerin fusions were cleaved overnight at 25°C with 50 μg His-tagged TEV protease (produced in-house) per mg of Fc-TEV-chemerin. Chemerin was purified with Protein A resin (Cytiva) and Ni-NTA resin (Qiagen, Hilden, Germany) to remove the cleaved monovalent Fc and His-tagged TEV protease, respectively. Preparative size exclusion chromatography (Superdex 75 pg, Cytiva) was performed to remove aggregation. Sample identity and purity were determined by QTOF mass spectrometry, aSEC-MALS and SDS-Page.

For binding experiments and functional assays, carrier-free human CCL2, CCL5, CCL7, CCL8, CCL19, CCL21, CXCL8 and murine CCL2, CCL5, CCL7, CCL19, CCL21 recombinant proteins were purchased commercially (R&D Systems, Minneapolis, MN). Carrier-free murine CCL8 was purchased from Biolegend (San Diego, CA). Anti-huCCRL2 and anti-muCCRL2 antibodies were purchased commercially (S1 Table). IgG2a isotype (Pfizer Inc., NY, NY) and IgG2b isotype (R&D Systems) were used as controls for K097F7 and 152254, respectively. IgG2a isotype (Pfizer Inc.) was used as a control for BZ5B8. For antibody binding assays, Rhodamine Red-X (RRX)-conjugated goat anti-mouse or anti-rat secondary antibodies (Jackson ImmunoResearch, West Grove, PA) were used to detect anti-CCRL2 primary antibodies.

### Flow cytometry binding assay

CCRL2 ligands were labeled with biotin using the EZ-Link Sulfo-NHS-LC-Biotin kit (ThermoFisher Scientific) following the manufacturer’s instructions. Briefly, 10 mM NHS-biotin stock was added to a final biotin-to-analyte molar ratio of 5:1 and the mixture was incubated at room temperature (RT) for 1 h prior to dialysis with phosphate buffered saline (PBS). Biotin-labeled ligands were confirmed by SDS-PAGE (S1 Fig). To assess ligand or antibody binding, HEK-huCCRL2, HEK-muCCRL2 or parental cells were seeded at 2x10^4 cells/well into 384-well plates. Serially diluted biotin-labeled analyte in PBS + 0.1% (w/v) bovine serum albumen (BSA) was then added to each well, incubated at 4°C for 45 min, then washed 2x with PBS + 0.1% (w/v) BSA. To detect cell bound biotin-labeled ligands, RRX-conjugated streptavidin (Jackson ImmunoResearch) was added, and cells were incubated at 4°C for 45 min. Cells were then washed 2x with PBS + 0.1% (w/v) BSA, labeled with DAPI (ThermoFisher Scientific), and analyzed by flow cytometry.

To identify antibodies that block chemerin binding, HEK-huCCRL2 or HEK-muCCRL2 cells were seeded in each well of a 384-well plate and incubated with 20 μl of serially diluted unlabeled anti-human or anti-mouse CCRL2 primary antibodies at 4°C for 45 min. Then, biotin-labeled chemerin was added to a final concentration of 10 nM and incubated at 4°C for 45 min. Cells were washed 2x with PBS + 0.1% (w/v) BSA, incubated with RRX-conjugated streptavidin at 4°C for 45 min, and unbound streptavidin was removed by washing 2x with PBS + 0.1% (w/v) BSA prior to flow cytometry analysis. GraphPad Prism (version 9.0) (GraphPad Software, Inc., La Jolla, CA) was used to calculate EC_50_ and IC_50_ values for flow cytometry binding experiments. EC_50_ values were determined by performing the log (agonist) vs. response variable slope (four parameter) equation. IC_50_ values for CCRL2 antibodies and chemerin in flow cytometry competition experiments were determined using the log (inhibitor) vs. response variable slope (four parameter) equation.

### SPRm

Wild-type HEK 293 cells were cultured in DMEM, 10% (v/v) FBS and HEK-huCCRL2 cells were cultured in DMEM, 10% (v/v) FBS supplemented with 1.75 mg/ml G418 (ThermoFisher Scientific). Ligand binding was quantified using the SPRm 200 Series (Biosensing Instrument, Phoenix, AZ). Briefly, 300 μl of live cells at 1x10^5 cells/ml were seeded on gold coated glass slides pre-coated with 50 μg/mL rat collagen type I (Corning Inc., Corning, NY) and incubated overnight at 37°C, 5% CO_2_. Prior to injecting ligand or antibody, SPRm 200 was primed 3x with running buffer consisting of PBS supplemented with 0.1% (w/v) BSA. Increasing concentrations of chemerin (10 nM, 26 nM, 64 nM, 160 nM, 400 nM) or CCRL2 antibodies (6 nM, 16 nM, 40 nM, 100 nM) were injected at 150 μl/min, with a contact time of 3 min and a dissociation time of 5 min. To assess binding of multiple analytes, cells were washed 5x 2 min between each run. Binding data were evaluated using Biosensing Instrument. Image Analysis Software (version 1.6.2) and were fitted to a 1:1 binding model.

### Neutrophil isolation from human subjects

Primary human blood samples were obtained with informed written consent from healthy human adult donors in accordance with Pfizer Inc. Global Occupational Health and Wellness Research Support Program (protocol GOHW RDP-01) approved by the Schulman Institutional Review Board. Peripheral blood was obtained by venipuncture and drawn into K2-EDTA vacutainer blood collection tubes (BD Biosciences, Franklin Lakes, NJ) prior to experimentation. Neutrophils were isolated using the MACSxpress Whole Blood Neutrophil Isolation Kit (Miltenyi Biotec, Bergisch Gladbach, Germany) following the manufacturer’s instructions. For neutrophil purification, red blood cells were depleted using the Erythrocyte Depletion Kit (Miltenyi Biotec) following the manufacturer’s instructions. Isolated neutrophils were washed 3x with the respective assay buffer to completely remove serum and EDTA.

### Neutrophil degranulation

Primary human neutrophil degranulation was assessed by measuring myeloperoxidase (MPO) secreted into the cell culture medium. Isolated neutrophils were resuspended in RPMI-1640 containing L-glutamine (ThermoFisher Scientific) and supplemented with 0.1% (w/v) fatty acid free BSA. 1x10^5 neutrophils were plated at a final volume of 100 μl in each well of a 96-well round bottom polypropylene plate (Corning Inc.) and pre-incubated with CCRL2 antibodies K097F7 (Biolegend), 152254 (R&D Systems) or isotype controls for 15 min. IgG2a isotype (Pfizer Inc.) and IgG2b isotype (R&D Systems) were used as controls for K097F7 and 152254, respectively. Neutrophils were then primed with 5 μg/ml cytochalasin B (Sigma-Aldrich, St. Louis, MO) for 15 min, followed by stimulation with 100 ng/ml of CXCL8 for 30 min. Plates were then centrifuged at 1000 rpm for 1 min and supernatants were collected. Supernatant MPO was quantified using the Human Myeloperoxidase (MPO) ELISA Kit (Mesoscale Discovery, Rockville, MD) following the manufacturer’s instructions. All incubation steps were performed at 37°C, 5% CO_2_.

### CXCL8 neutrophil peritoneal model

All activities involving laboratory animals were carried out in accordance with federal, state, local, and institutional guidelines governing the use of laboratory animals in research and were reviewed and approved by Pfizer Institutional Animal Care and Use Committee. Pfizer animal care facilities that supported this work are fully accredited by AAALAC International.

Female C57BL/6 mice (7–10 weeks old, Jackson Laboratory, Bar Harbor ME) were injected with 300 μl of PBS containing BZ5B8 anti-CCRL2 antibody (Absolute Antibody, Oxford, United Kingdom) at indicated concentrations (30 μg, 100 μg or 300 μg) or isotype control (Pfizer Inc.) intraperitoneally (IP). Twenty hours later, animals were injected IP with 200 μl of either PBS alone or 300 μg CXCL8 (R&D Systems) reconstituted in PBS to induce neutrophil recruitment to the peritoneum. Animals were euthanized 4 h later (24 h post antibody treatment) with CO_2_ and peritoneal lavages were performed with 3 ml of PBS supplemented with 10% (v/v) FBS (ThermoFisher Scientific). Leukocytes in lavage fluid were then assessed by flow cytometry. To determine terminal antibody plasma concentrations, 10 μl of blood was collected via tail vein immediately prior to euthanasia and diluted in 190 μl exposure buffer (0.2 M Tricine, 0.15 M NaCl, 3 mM EDTA, 1% (w/v) BSA, 0.1% (v/v) Tween 20, pH 8.5) prior to analysis.

For flow cytometry analyses of peritoneal cell types, 1 ml of peritoneal lavage suspension was centrifuged at 300 g at 4°C for 5 min, then resuspended in 100 μl of autoMACS rinsing solution with BSA (Miltenyi Biotec). Cells were incubated with anti-CD16/CD32 for 15 min on ice to block Fc receptors, then stained with a cocktail of antibodies (S2 Table) for 30 min at 4°C. One hundred microliters of Brilliant Stain Buffer (BD Biosciences) were used during antibody staining for all samples. Cells were washed twice and resuspended in staining buffer (PBS, 0.5% BSA, 2 mM EDTA) for flow cytometry. 7-AAD viability dye was used for live/dead cell discrimination.

### Determination of mouse anti-CCRL2 antibody plasma concentration

Anti-CCRL2 antibody BZ5B8 concentrations were determined in mouse plasma (samples centrifuged from diluted whole blood) using a qualified ligand binding assay on an ELISA platform (Mesoscale Discovery). Briefly, a CCRL2 N-terminal peptide consisting of amino acids 1–24 (Bio-Synthesis Inc., Lewisville, TX) was coated onto an MSD Multi-Array^TM^ plate, the plate was blocked, and mouse anti-CCRL2 antibody was captured onto the blocked plate by the CCRL2 peptide. Bound anti-CCRL2 antibody was detected using a ruthenylated goat anti-mouse IgG. Plates were read on an MSD QuickPlex Imager and final detection used the ruthenium-labeled antibody and tripropylamine to produce an electrochemiluminescent signal representative of the amount of bound mouse anti-CCRL2 antibody. Sample concentrations were determined by interpolation from a standard curve that was fitted using a 4-parameter logistic equation with 1/y^2^ response weighting. The detection range in 100% C57BL/6 mouse plasma was 0.080 to 5.12 μg/ml.

### Profiling of CCRL2 expressing leukocyte populations by flow cytometry

Human peripheral blood was collected in K2-EDTA vacutainer blood collection tubes (BD) from healthy donors as described above for neutrophil isolation methods. Whole blood was stained with a cocktail of antibodies (S3 Table) with Brilliant Stain Buffer Plus (BD Biosciences) for 30 min at RT. To perform flow cytometry on isolated neutrophils, neutrophils were isolated from human peripheral blood using the EasySep^™^ Direct Human Neutrophil Isolation Kit (StemCell Technologies) and Fc blocked (BD Biosciences) for 5 min at 4°C prior to staining. For competitive binding experiments, neutrophils were pre-incubated with 20x unlabeled anti-CCRL2 antibody for 30 min at 4°C, then immediately stained with anti-CD45, anti-CD66b, anti-CD15, and PE-conjugated anti-CCRL2 or isotype control with Brilliant Stain Buffer Plus (BD Biosciences) for 30 min at 4°C (S3 Table). Following staining, cells were washed with Pharmingen Stain Buffer with BSA (BD Biosciences) and fixed in 1.75% (v/v) formaldehyde. Samples were collected on a FACSymphony A5 flow cytometer (BD Biosciences).

For whole blood immunophenotyping: CD45 was used for live cell gating followed by classical SSC/FSC gates. Doublets were removed based on FSC-H/FSC-A. Neutrophils were defined as CD66b^+^, CD15^+^, SSC^hi^, CD16^+^. Monocytes were defined as CD66b^-^, CD15^-^, CD14^+^, CD19^-^, CD3^-^, and subclassified as classical monocytes by CD14^++^, CD16^-^; intermediate monocytes by CD14^++^, CD16^+^; and non-classical monocytes by CD14^+^, CD16^+^. B cells were defined as CD66b^-^, CD15^-^, CD14^-^, CD19^+^, CD3^-^, CD20^+^. Dendritic cells were defined as CD66b^-^, CD15^-^, CD14^-^, CD19^-^, CD3^-^, CD56^-^, HLA-DR^+^ and subclassified as myeloid DCs by CD11c^+^, CD123^-^; immature DCs CD11c^+^, lin^-^, CD33^lo^; and plasmacytoid DCs as CD11c^-^, CD123^+^, CD303^+^. NK cells were defined as CD66b^-^, CD15^-^, CD14^-^, CD19^-^, CD3^-^, CD56^+^, CD123^-^, CD16^+^/^-^. NKT cells were defined as CD66b^-^, CD15^-^, CD14^-^, CD19^-^, CD3^+^, CD56^+^. T cells were defined as CD66b^-^, CD15^-^, CD14^-^, CD19^-^, CD3^+^, CD56^-^, and differentiated based on CD4^+^ and CD8^+^ markers.

### Immunophenotyping of CCRL2 flow cytometry panel using OMIQ software

Flow Cytometry Standard (FCS) files were extracted from the BD FACSymphony A5 flow cytometer and uploaded into OMIQ software. Sample data were compensated, scaled, normalized and manually gated based on the described immunophenotyping panel reported in the profiling of CCRL2 leukocyte populations by flow cytometry section of Materials and methods. FCS files from four individual donors and one CCRL2 PE Fluorescence Minus One (FMO) control were subsampled to collect a maximum of 100k events in each respective CD45+ backgated population, for a total of 500k events. Events were run through a UMAP dimension reduction algorithm with the following settings: (neighbors: 15, minimum distance: 0.4, components: 2, metric: euclidean, learning rate: 1, epochs: 200, random seed: 4381, embedding initialization: spectral). Resulting UMAP clustering was set to display relative CCRL2 PE intensity across clustered populations. Finally, manually gated cell populations were mapped onto UMAP to identify clustered populations that correlate with the PE intensities in donor samples and FMO control.

### Statistical analysis

Statistical analyses and data graphing were performed using GraphPad Prism (version 9.0). Statistical analyses were performed on in vivo flow cytometry profiling results. To compare peritoneal cell populations and surface receptor expression between PBS and CXCL8 groups in vivo, statistical significance was calculated using an unpaired t-test with Welch’s correction. To compare peritoneal cell populations and surface marker expression following CXCL8 stimulation between isotype control antibody groups and groups dosed with BZ5B8, a Brown-Forsythe and Welch ANOVA test with Dunnett’s T3 multiple comparisons test was performed. All statistical analyses were performed with an alpha threshold of 0.05. Data are presented as Mean ± SEM unless otherwise noted.

## Results

### Chemerin binds human and murine CCRL2

**Fig 1:**
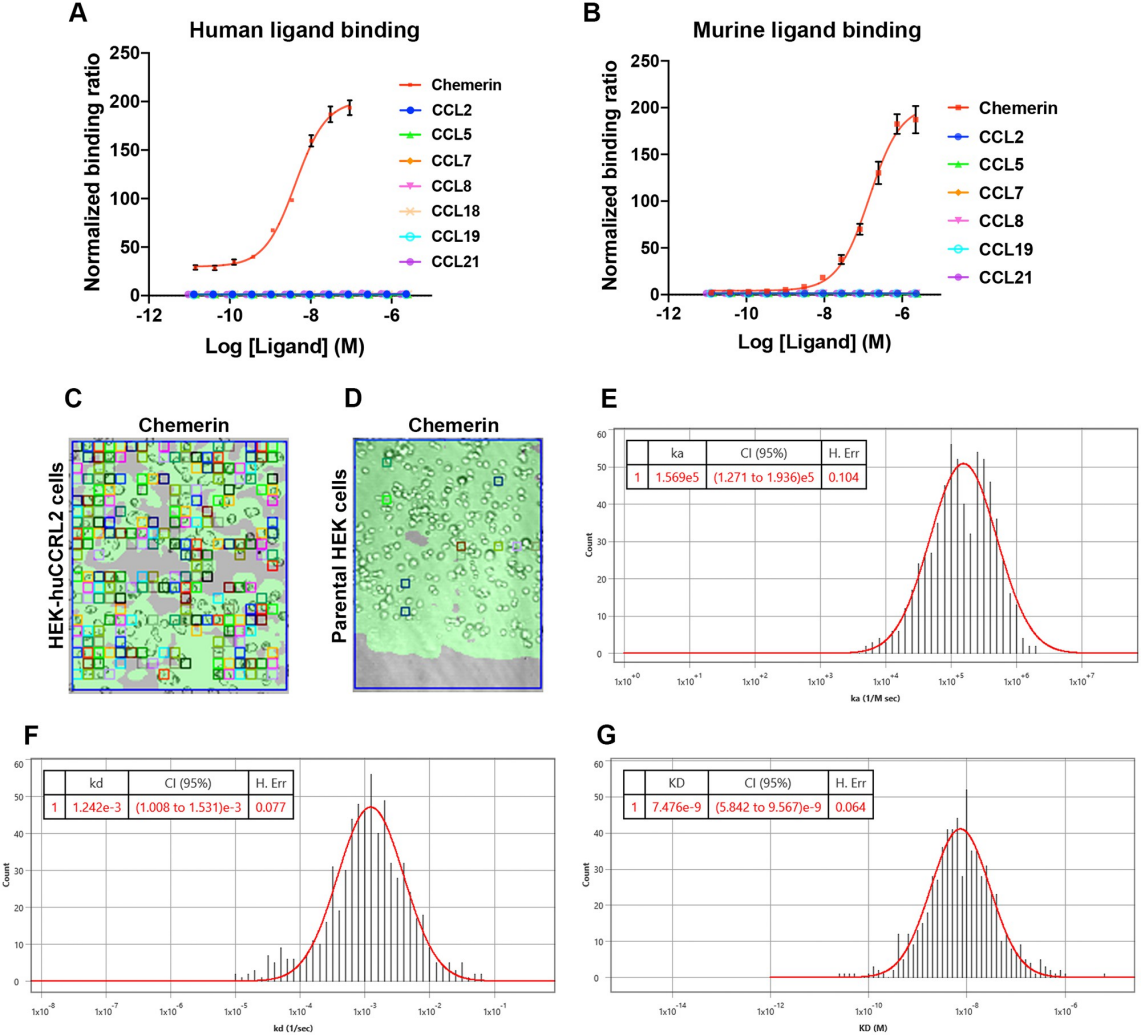
Chemerin binds cell surface expressed CCRL2. (A) Biotin-labeled human ligands were incubated with HEK-huCCRL2 cells and (B) biotin-labeled murine ligands were incubated with HEK-muCCRL2 cells for flow cytometry binding experiments. Ligand binding is shown as median fluorescence intensity (MFI) of ligand bound to HEK-CCRL2 cells normalized to parental HEK cell binding, Mean ± SEM (N = 3) for (A) and Mean ± SEM (N≥5) for (B). EC_50_ values were determined by performing the log (agonist) vs. response variable slope (four parameter) equation using GraphPad Prism (version 9.0). SPRm brightfield image of (C) human chemerin bound to HEK-huCCRL2 cells or (D) human chemerin bound to parental HEK cells; squares indicate ROIs where chemerin binding events were detected and are representative of N = 3. Representative histogram distributions for (E) on-rate (k_on_), (F) off-rate (k_off_) and (G) binding affinity (K_D_) were generated from kinetic fit of ROI sensorgrams for human chemerin binding to HEK-huCCRL2 cells and are representative experiments of N = 3. Histograms of on-rate (E) and off-rate (F) values generated using Biosensing Instrument Image Analysis Software (version 1.6.2) are assigned units of ka (1/M sec) and kd (1/sec), respectively, but are referred to as k_on_ (M^-1^ s^-1^) (on-rate) and k_off_ (s^-1^) (off-rate) elsewhere in this manuscript according to conventional nomenclature.

CCRL2 is reported to bind several ligands including chemerin, CCL2, CCL5, CCL7, CCL8, CCL18, CCL19, and CCL21 [9–13]. Among these ligands, chemerin is the most widely studied for CCRL2 [8, 12, 14, 23]. To inform our efforts to identify antibodies that block CCRL2 ligand binding function, we first set out to ascertain the ligands that directly bind CCRL2. We generated HEK-human (hu) CCRL2 cells and HEK-murine (mu) CCRL2 cells that stably overexpress human and mouse CCRL2, respectively. Using these cells, we developed a flow cytometry assay to detect biotin-labeled ligand binding to CCRL2. To assess ligand quality post biotin-labeling, we confirmed that each biotin-labeled ligand produces a single band as expected when analyzed on reducing and non-reducing SDS gels (S1 Fig). Human and murine chemerin specifically bound HEK-huCCRL2 cells (EC_50_ = 4.2 nM) and HEK-muCCRL2 cells (EC_50_ = 142.6 nM) in a dose-dependent manner, respectively (Fig 1A and 1B), as illustrated in the normalized plot against parental HEK cell binding. No specific binding of human and murine CCL2, CCL5, CCL7, CCL8, CCL19 and CCL21 to the respective CCRL2 overexpression cell line was detected (Fig 1A and 1B). Mice do not express CCL18 [24], therefore, only human CCL18 was tested with HEK-huCCRL2 cells and no specific binding was observed (Fig 1A).

To understand the binding kinetics of chemerin and exclude potential labeling artifact, we implemented a live-cell SPRm methodology to detect binding of label-free chemerin to HEK-huCCRL2 cells. SPRm incorporates SPR and brightfield microscopy to detect binding of unlabeled molecules to cell surface targets (S2A Fig). Similar to conventional biosensor SPR technologies (such as Biacore), rate kinetics and binding affinity can be determined [25]. SPRm uniformly divides the sensor surface into hundreds of regions of interest (ROIs) and ROIs that do not detect a binding response are filtered out by the analysis software. ROIs are depicted as multicolored squares on SPRm images. ROI color difference does not reflect magnitude of binding response of a particular region, rather is randomly assigned by the analysis software for visualization purposes only. Numerous ROIs were observed and overlapped cells when chemerin was incubated with HEK-huCCRL2 cells (Fig 1C), but few ROIs were present when chemerin was incubated with parental HEK cells (Fig 1D), indicating that chemerin specifically binds cells expressing CCRL2. Kinetic data from a single representative experiment assessing chemerin binding to HEK-huCCRL2 cells show robust gaussian distributions for calculated on-rate (Fig 1E), off-rate (Fig 1F) and binding affinity (Fig 1G), whereas minimal binding of chemerin to parental HEK cells was detected and K_D_ values could not be calculated (S2B Fig). The mean values for chemerin binding HEK-huCCRL2 cells across all experiments performed for on-rate (k_on_ = 3.11E+05 M^-1^ s^-1^), off-rate (k_off_ = 1.16E-03 s^-1^) and binding affinity (K_D_ = 5.49 nM) are reported in Table 1, and are consistent with biotin-chemerin binding to HEK-huCCRL2 cells (Table 1). Importantly, our mean K_D_ of chemerin binding to huCCRL2 determined by SPRm was comparable to our flow cytometry results (Fig 1A) as well as a previously reported binding affinity (2.35 nM) determined by radiolabeled chemerin binding to cells overexpressing huCCRL2 [26]. To investigate whether biotin-labeling may interfere with ligand binding to HEK-huCCLR2 cells (Fig 1A), a subset of label-free C-C chemokines was assessed by SPRm. Few ROIs were present when label-free CCL2 and CCL5 were incubated with HEK-huCCRL2 cells and K_D_ values could not be calculated (S3 Fig), corroborating Fig 1A.

**Table 1:** Summary of human CCRL2 antibody and chemerin binding to HEK-huCCRL2 cells using live-cell SPRm (n = 3).

Ligand	k_on_ (M^-1^ s^-1^) ± SD	k_off_ (s^-1^) ± SD	K_D_ (nM) ± SD
chemerin	3.11E+05 ± 2.4E+05	1.16E-03 ± 4.8E-04	5.49 ± 4
Biotin-chemerin	2.97E+05 ± 2.7E+05	1.84E-03 ± 9.5E-04	8.55 ± 6
K097F7	5.65E+05 ± 4.2E+05	8.28E-04 ± 4.4E-05	2.48 ± 2
152254	5.88E+05 ± 4.2E+05	1.94E-03 ± 6.5E-04	4.75 ± 4

SD: Standard deviation.

### Identification and characterization of CCRL2 neutralizing antibodies

**Fig 2:**
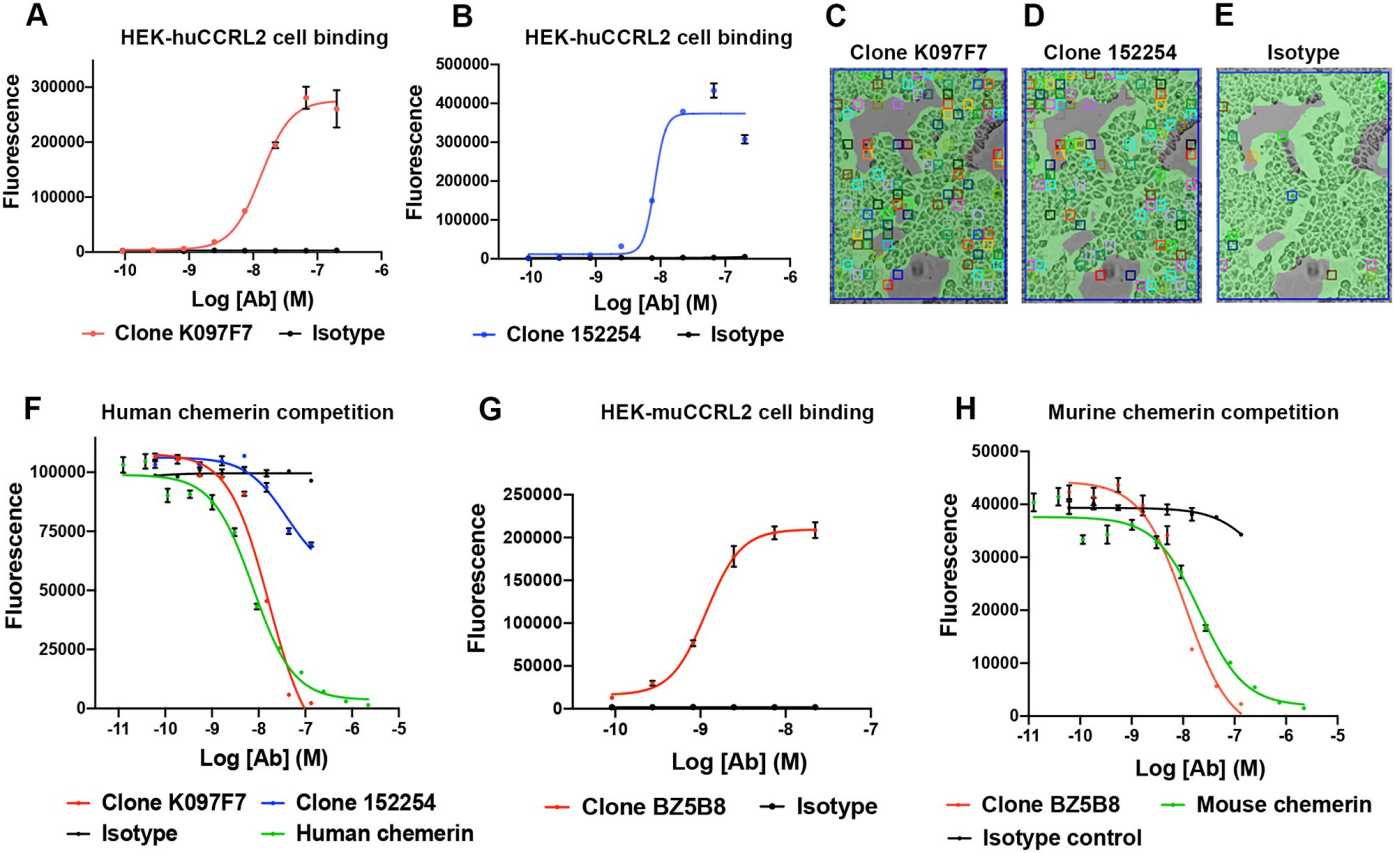
Binding and chemerin competition of human and murine CCRL2 antibodies. Flow cytometry detection of CCRL2 antibodies binding to HEK-huCCRL2 cells was performed with (A) K097F7 and (B) 152254, and binding is shown as MFI Mean ± SEM (N = 3). Representative SPRm brightfield images of (C) K097F7 binding to HEK-huCCRL2 cells, (D) 152254 binding to HEK-huCCRL2 cells, or (E) isotype control binding to HEK-huCCRL2 cells; squares indicate ROIs where antibody binding events were detected. (F) K097F7, 152254, unlabeled human chemerin or isotype control were incubated with HEK-huCCRL2 cells prior to addition of 10 nM biotin-labeled human chemerin to assess antibody competition of ligand binding to CCRL2 Mean ± SEM (N = 3). (G) Anti-muCCRL2 BZ5B8 or isotype control was incubated with HEK-muCCRL2 cells and binding is shown as MFI Mean ± SEM (N = 3). (H) BZ5B8, unlabeled murine chemerin or isotype control were incubated with HEK-huCCRL2 cells prior to addition of 10 nM biotin-labeled murine chemerin and binding is shown as MFI Mean ± SEM (N = 3). EC_50_ values were determined by performing the log (agonist) vs. response variable slope (four parameter) equation and IC_50_ values were determined by performing the log (inhibitor) vs. response variable slope (four parameter) equation using GraphPad Prism (version 9.0).

As a non-signaling GPCR, CCRL2 does not activate intracellular G proteins nor recruit β-Arrestins upon ligand binding [26]. Rather, CCRL2 concentrates its ligand at the plasma membrane [23]. Given the lack of known downstream signaling, identifying tools that neutralize CCRL2 from binding its ligand is key to studying the function of CCRL2. To enable these efforts, we demonstrated that two anti-huCCRL2 antibody clones, K097F7 and 152254, specifically bound to HEK-huCCRL2 cells in our flow cytometry assay (K097F7 EC_50_ = 13.29 nM; 152254 EC_50_ = 8.13 nM) (Fig 2A and 2B). As a confirmatory method of antibody binding, SPRm was performed with K097F7 and 152254, and numerous ROIs were detected on HEK-huCCRL2 cells (Fig 2C and 2D), but not on parental HEK cells (S2D and S2F Fig). The binding affinity (K_D_) for K097F7 and 152254 to HEK-huCCRL2 cells was 2.48 nM and 4.75 nM, respectively (Table 1). Importantly, minimal binding of K097F7 and 152254 to parental HEK cells resulted in K_D_ values that could not be calculated (S2D and S2F Fig). Moreover, minimal binding of isotype control to HEK-huCCRL2 cells was observed (Fig 2E), indicating that K097F7 and 152254 bound specifically to CCRL2.

Next, we sought to determine whether K097F7 or 152254 neutralized the ligand binding function of CCRL2. K097F7 completely neutralized biotinylated chemerin binding to human CCRL2 dose-dependently (IC_50_ = 16.64 nM) compared to incomplete inhibition observed at high concentrations with 152254 (Fig 2F). As a positive control, unlabeled chemerin inhibited biotinylated chemerin binding to HEK-huCCRL2 cells (IC_50_ = 7.55 nM) at a comparable potency to K097F7 (Fig 2F). Given that human and mouse CCRL2 amino acid sequences only share 51% sequence identity [23], identifying antibodies that are human-mouse cross-reactive is challenging. Therefore, we profiled 3 anti-murine CCRL2 antibodies to identify a surrogate antibody for muCCRL2 with ligand binding neutralizing activity for in vivo studies. Binding of antibodies BZ5B8, 11n20 and 498321 to HEK-muCCRL2 cells was confirmed (Fig 2G, S4A and S4B Fig). Interestingly, only BZ5B8 neutralized the ligand binding function of muCCRL2 (IC_50_ = 11.2 nM) (Fig 2H), whereas 11n20 and 498321 promoted chemerin binding (S4C Fig). As a positive control, unlabeled murine chemerin inhibited biotinylated murine chemerin binding CCRL2 with an IC_50_ of 21.1 nM (Fig 2H), demonstrating the sensitivity of the assay system.

### CCRL2 ligand binding neutralizing antibodies do not reduce CXCL8 induced neutrophil activation in vitro

**Fig 3:**
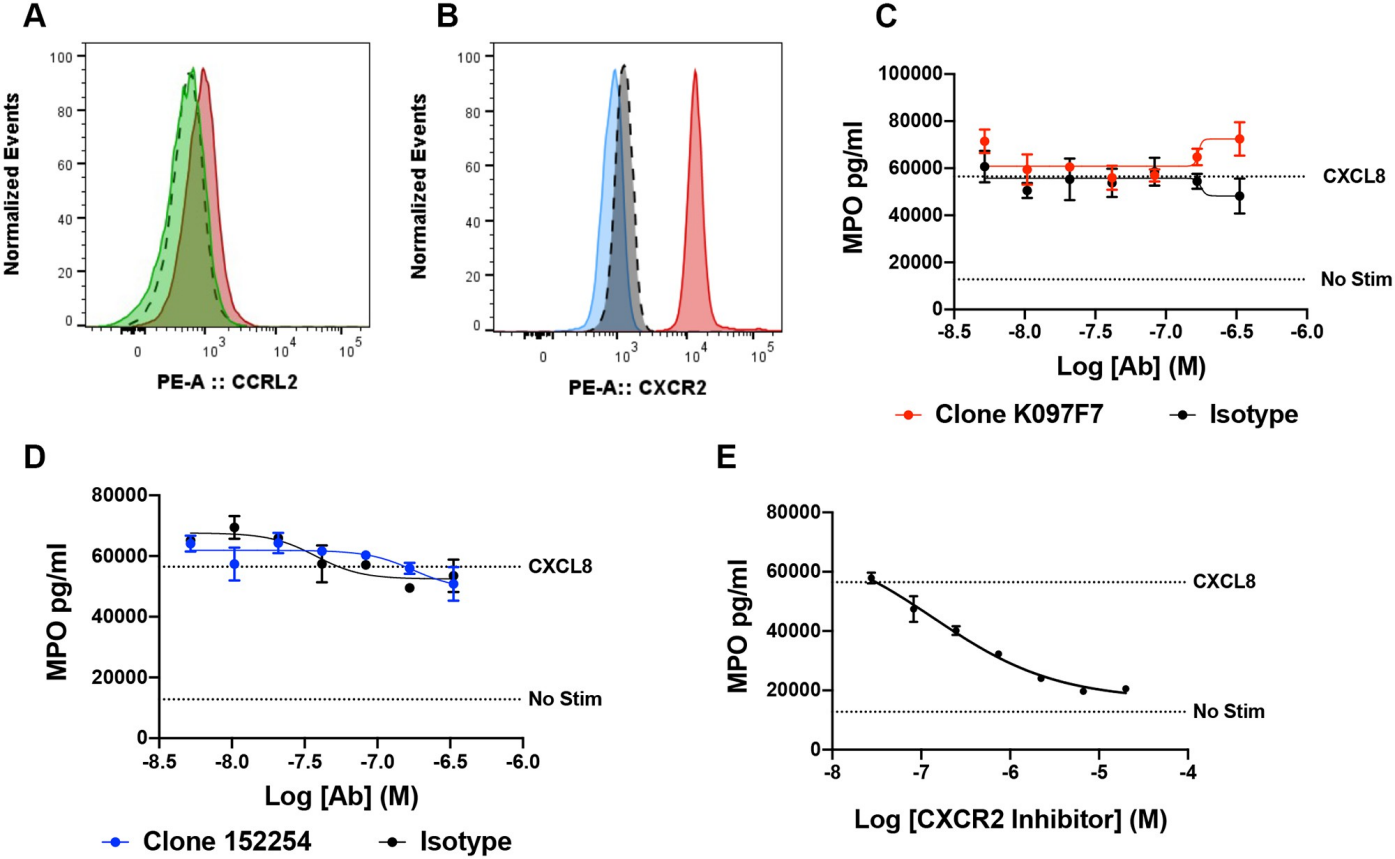
Effect of CCRL2 neutralization on primary human neutrophil degranulation. (A) Representative histogram quantifying CCRL2 expression on isolated primary human neutrophils using PE-conjugated K097F7 (red) and is representative of neutrophils profiled from 4 human donors. CCRL2 specific staining was determined by incubating neutrophils with unlabeled K097F7 prior to addition of PE-labelled K097F7 (green) and was compared to isotype control staining (black segmented). (B) Representative histogram of CXCR2 expression on isolated primary human neutrophils using PE-conjugated anti-CXCR2 (red) and is representative of neutrophils profiled from 3 human donors. CXCR2 staining was compared to PE-isotype control staining (grey dotted line) and unstained control (blue) neutrophils. (C-E) Degranulation of primary neutrophils. Primary neutrophils were pre-incubated with (C) K097F7, (D) 152254, or (E) the CXCR2 small molecule inhibitor SB225002, then stimulated with 100 ng/ml CXCL8. Supernatant concentrations of MPO were quantified by ELISA. (C-E) Shown are representative data from a single donor which are presented as Mean ± SEM (N = 2) (a total of 3 different neutrophil donors were tested independently).

CCRL2 has been shown to form a heterodimer with CXCR2 and regulate CXCL8 signaling in neutrophil activation and migration [17]. Specifically, neutrophils isolated from CCRL2 deficient mice exhibited reduced degranulation in response to CXCL8 compared to wild-type mice [17]. To interrogate a CCRL2/CXCR2 axis in human neutrophils, we first confirmed that CCRL2 and CXCR2 are expressed on the surface of isolated primary human neutrophils (Fig 3A and 3B). We next tested whether K0970F7, which neutralized CCRL2 (Fig 2F), modulated neutrophil degranulation in response to CXCL8 by detecting neutrophil myeloperoxidase (MPO) secreted into the supernatant. MPO is stored in primary azurophilic granules and catalyzes the formation of reactive oxygen intermediates [27, 28]. Additionally, MPO is released into the extracellular space during degranulation, or exocytosis of granule components, in response to pathogenic or inflammatory stimuli [28, 29]. Despite different neutralization profiles, neither K097F7 nor 152254 attenuated MPO release, as was also observed with their respective isotype controls (Fig 3C and 3D). As a positive control, inhibition of CXCR2 with the small molecule antagonist SB225002 dose-dependently inhibited CXCL8-induced MPO release from neutrophils, indicating that the neutrophil activation observed in this assay was indeed mediated by activation of CXCR2 (Fig 3E).

### CCRL2 ligand binding neutralizing antibody does not inhibit CXCL8 induced neutrophil and myeloid cell recruitment in vivo

**Fig 4:**
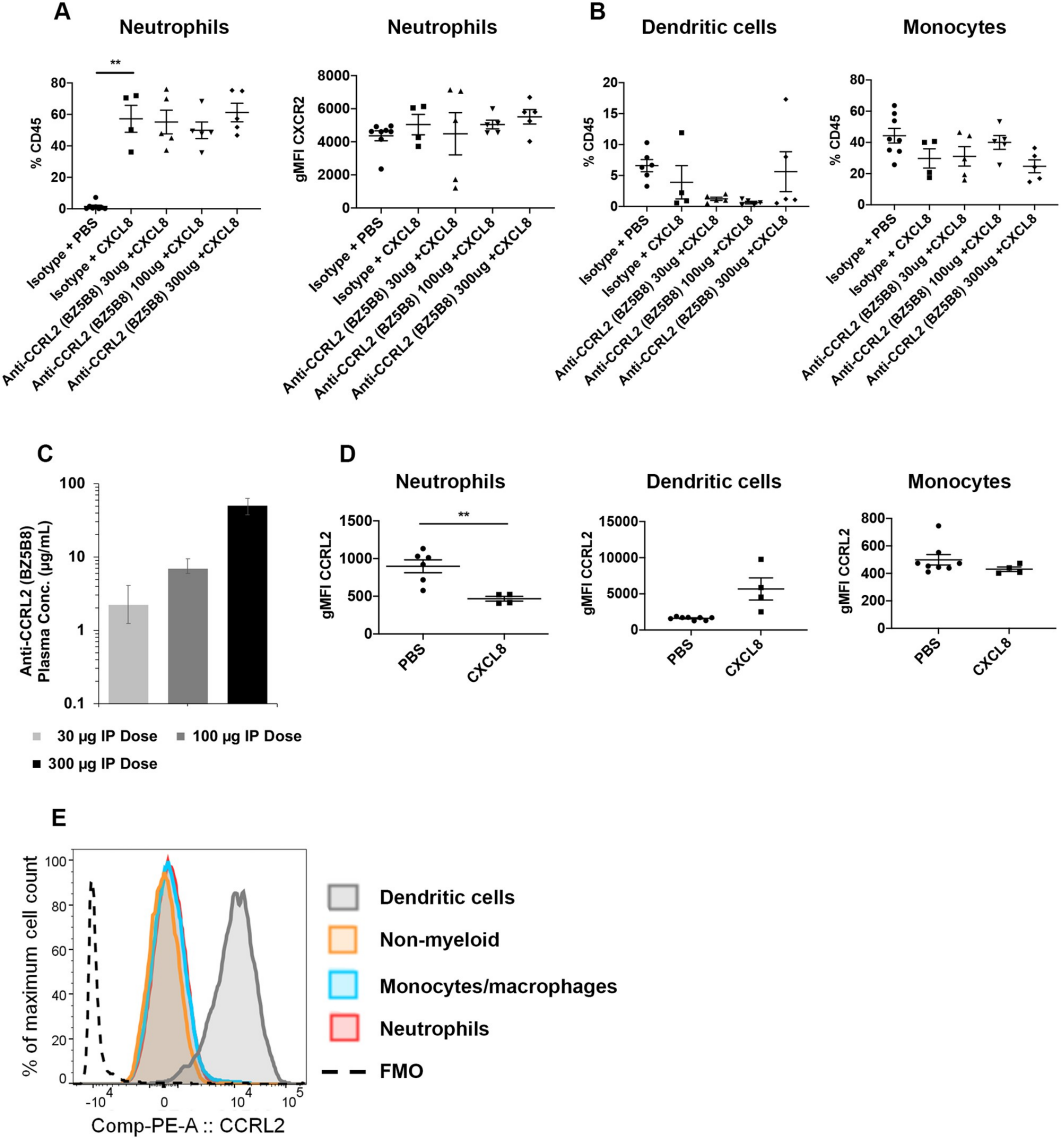
Flow cytometric analysis of peritoneal leukocytes populations following BZ5B8 administration in vivo. Female C57BL/6 mice were injected with anti-CCRL2 antibody BZ5B8 or isotype control intraperitoneally (IP), followed by human CXCL8 or PBS IP injection to induce the model. Peritoneal lavages were harvested 4 h later for flow cytometry analysis. (A) Percentages of neutrophils (*left)* out of total CD45+ leukocytes in peritoneal lavages and surface expression of CXCR2 on neutrophils (*right*) are reported for each treatment group. (B) Percentages of dendritic cells *(left)* and monocytes *(right)* out of total CD45+ leukocytes in peritoneal lavages are reported for each treatment group. (C) Mouse anti-CCRL2 antibody BZ5B8 plasma concentration (Mean ± SD) 24 h after IP dose. (D) CCRL2 expression is shown on neutrophils (*left)*, dendritic cells *(middle)* and monocytes *(right)* in control mice stimulated with CXCL8 or PBS control. (E) Representative histogram of CCRL2 expression on dendritic cells (grey), non-myeloid cells (CD11b-, Ly6G-) (orange), monocytes/macrophages (blue) or neutrophils (red) compared to FMO control in peritoneal lavages from CXCL8 stimulated mice. (A-B, D) Statistical analyses were performed using the Brown-Forsythe and Welch ANOVA with Dunnett’s T3 multiple comparisons test, and a unpaired t test with Welch’s correction. Statistical analyses used an alpha threshold of 0.05. Results are plotted as the Mean ± SEM. **P<0.01.

CXCL8 is a potent neutrophil chemoattractant that directs neutrophil migration to sites of inflammation by activation of CXCR2 and this activity is reported to be regulated through CCRL2/CXCR2 heterodimer formation [17]. To investigate this mechanism in vivo using a neutralizing antibody approach, we first established a peritoneal neutrophil recruitment model. We detected a significant increase in neutrophil migration following i.p. injection of CXCL8 (Fig 4A), consistent with previous reports using CXCL8 as a stimulus in vivo [17, 30]. Given our results demonstrating that BZ5B8 neutralized CCRL2 in vitro (Fig 2H), we interrogated the impact of CCRL2 neutralization on neutrophil recruitment in vivo using this antibody. BZ5B8 did not inhibit neutrophil recruitment to the peritoneum following CXCL8 stimulation (Fig 4A), which differs from published data with CCRL2 deficient mice [17]. In humans, CXCL8 induces rapid internalization of neutrophil CXCR2 and CCRL2 deficient murine neutrophils express higher levels of CXCR2 [17, 31]. As such, we monitored the surface expression of neutrophil CXCR2 across dose groups and found that CXCR2 expression was unchanged with CXCL8 treatment or BZ5B8 administration (Fig 4A).

In addition to neutrophils, previous studies have demonstrated that CCRL2 regulates DC and monocyte migration during acute inflammatory models [16, 32]. To test if neutralization of CCRL2 modulates DC and monocyte migration in a CXCL8-induced acute inflammatory model, we quantified DCs and monocytes in the peritoneum. A trending but not significant decrease in DCs and monocytes in the peritoneum was observed following CXCL8 stimulation compared to PBS alone (Fig 4B). Similar to the observed effect on neutrophils, BZ5B8 did not significantly modulate CXCL8-induced DC or monocyte migration when compared to the isotype control group (Fig 4B). To rule out inadequate BZ5B8 antibody target coverage confounding our interpretation of results, BZ5B8 concentrations were assessed for every animal 24 hours post dose and concentrations were found to be typical for an IgG antibody in mice [33]. Exposures increased with increasing dose supporting that antibody concentrations were sustained throughout the peritoneal model (Fig 4C).

Given that CCRL2 upregulation has been noted in neutrophils, monocytes and DCs in response to inflammatory stimuli [8, 16, 17], we quantified the expression of CCRL2 on neutrophils, monocytes and DCs isolated from peritoneal lavages to determine if CXCL8 regulates CCRL2 expression (Fig 4D). Interestingly, we observed a significant decrease in the level of CCRL2 on neutrophils in the peritoneum following CXCL8 administration compared to PBS controls (Fig 4D). Moreover, a trending but not significant increase in DC CCRL2 expression (P = 0.07) was noted in animals stimulated with CXCL8 compared to controls, whereas the levels of CCRL2 on monocytes was not modulated by CXCL8 (Fig 4D). A direct comparison of CCRL2 expression levels on leukocytes from peritoneal lavage of CXCL8 stimulated mice indeed confirmed that DCs more highly expressed CCLR2 than neutrophils, monocytes, or non-myeloid cells (Fig 4E).

### Profiling of CCRL2 expression on human whole blood leukocytes

**Fig 5:**
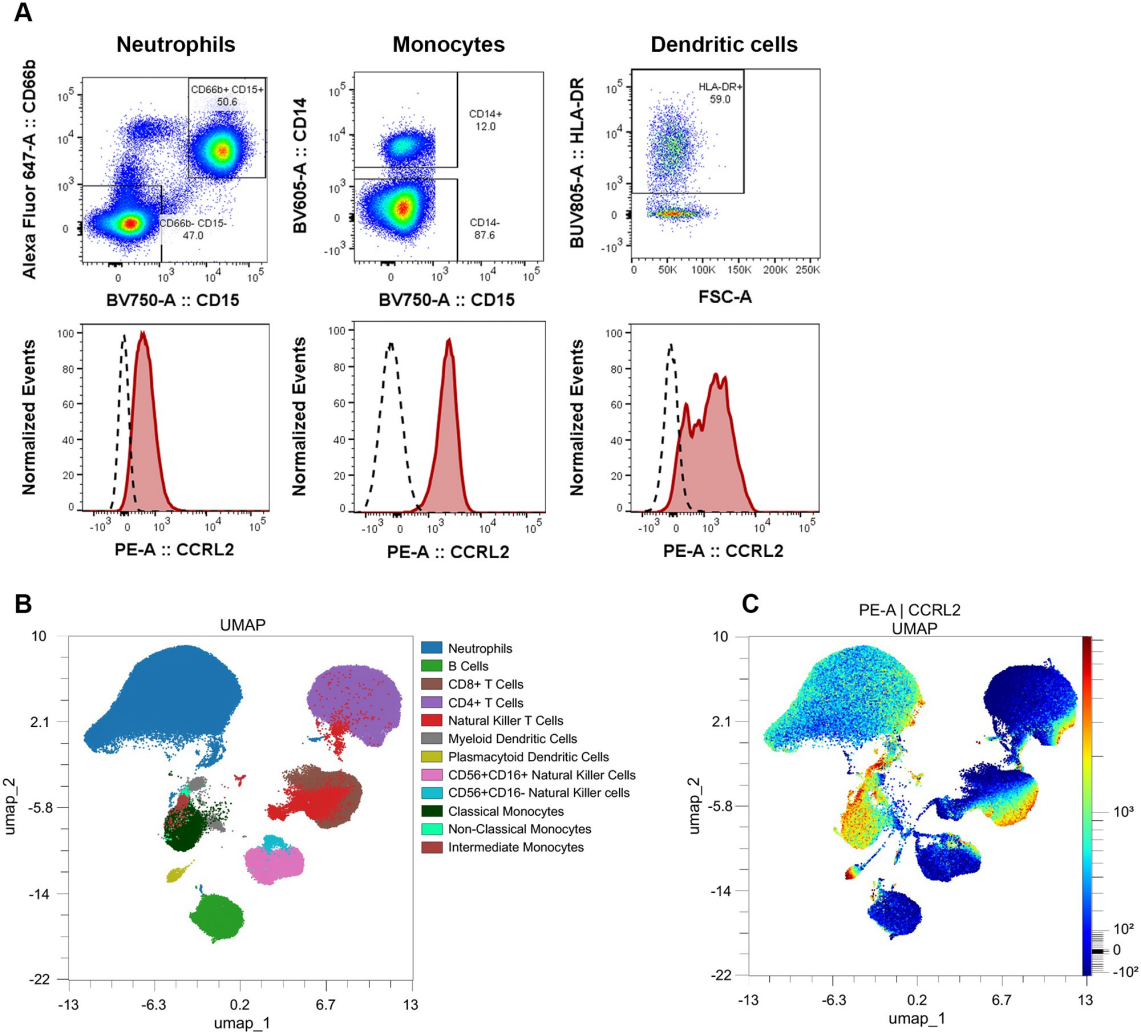
CCRL2 expression in whole blood leukocyte profiling. (A) Gating for neutrophils *(left)*, monocytes *(middle)* and dendritic cells *(right)* in whole blood flow cytometry analysis is depicted. Expression of CCRL2 on neutrophils *(left)*, monocytes *(middle)* and dendritic cells *(right)* is shown in red compared to FMO controls (black segmented). Data are representative of 4 donors. (B) Manually gated cell populations overlaid onto clustered UMAP populations show 12 leukocyte populations. Leukocyte subsets were defined using the gating strategy outlined in Materials and methods and S5 Fig. (C) CCRL2 expression within each leukocyte population was determined using relative PE intensity overlaid onto the same clustered populations of the UMAP in B.

To facilitate research on the function of CCRL2, we used an 18-antibody flow cytometry panel (S3 Table) to profile CCRL2 expression on 12 human peripheral blood leukocyte populations using the manual gating strategy outlined in S5 Fig. Neutrophils, monocytes and DCs expressed CCRL2 (Fig 5A), corroborating CCRL2 expression detected on isolated human neutrophils (Fig 3A) and CCRL2 profiling in vivo (Fig 4D and 4E). Of note, monocytes and DCs more highly expressed CCRL2 than neutrophils (Fig 5A). We then performed uniform manifold approximation and projection (UMAP) dimensional reduction analysis to visualize these populations (Fig 5B) and plotted the relative expression of CCRL2 for each population (Fig 5C). Uniform CCRL2 expression was detected in the neutrophil cluster (Fig 5B and 5C). CCRL2 has been detected in B cells in a maturation-dependent manner [34], however, this paradigm has been challenged by studies that did not detect B cell CCRL2 expression at steady state [35] or in vivo [36]. In our analysis of human leukocytes, the majority of B cells had little to no expression of CCRL2, with the exception of two small subclusters where CCRL2 expression was detected (Fig 5B and 5C). To confirm T cell expression of CCRL2 [35], T cells were clustered into distinct populations based on CD4 or CD8 expression (Fig 5B). While nearly half of CD8+ T cells expressed CCRL2, we noted that CCRL2 expression was detected only in a small subset of CD4+ T cells (Fig 5B and 5C). Individual UMAPs for CCRL2 expression in CD4+ T cell and CD8+ T cell populations confirmed this expression pattern (S6A Fig).

To our knowledge, CCRL2 expression has not been reported on Natural Killer T (NKT) cells. Two distinct populations of NKT cells were identified in our analyses and these populations clustered with CD4+ T cells and CD8+ T cells, respectively (Fig 5B). We plotted CD4+ T cell, CD8+ T cell and NKT cell populations individually to separate NKT cell CCRL2 expression from CCRL2 expression in CD4+ and CD8+ T cell subsets (S6A Fig). Approximately half of the subpopulation of NKT cells that clustered with CD8+ T cells expressed CCRL2, whereas minimal expression of CCRL2 was detected on NKT cells clustering with CD4+ T cells (Fig 5B and 5C, S6A Fig).

CCRL2 is expressed in monocytes and monocyte-derived dendritic cells in a maturation-dependent manner and CCRL2 promotes DC trafficking to lymph nodes in vivo [16, 35]. However, the expression and function of CCRL2 on primary human monocyte and DC subsets is not fully understood. We found that classical, non-classical and intermediate monocytes clustered closely (Fig 5B) and expressed CCRL2 (Fig 5C). We next profiled CCRL2 expression on myeloid DC (mDC) and plasmacytoid DC (pDC) subsets (Fig 5B and 5C). Since, mDCs clustered closely with monocyte populations (Fig 5B), we plotted individual CCRL2 UMAPs for DCs (S6B Fig) and monocytes (S6C Fig) to confirm subset specific expression of CCRL2. Non-classical and intermediate monocytes were less abundant than classical monocytes (S6C Fig) [37], but clustered together and highly expressed CCRL2 (Fig 5B and 5C, S6C Fig). High expression of CCRL2 was observed on both mDCs and pDCs, with pDCs clustering as a distinct population from mDCs and the monocyte subsets (Fig 5B and 5C, S6B Fig).

## Discussion

The best characterized function of CCRL2 is to bind and present ligands to shape chemokine gradients [8, 38]. To clarify the discrepancies with the reported ligands for CCRL2 [12, 23, 26], we used a combination of biotinylated and unlabeled ligand-receptor binding approaches to demonstrate that chemerin, but not the C-C chemokines CCL2, CCL5, CCL7, CCL8, CCL19 and CCL21 [9–12], directly binds human and murine CCRL2 with high affinity. Biotinylation can occur at any reactive primary amine, and SPRm assessment of chemerin binding to HEK-huCCRL2 cells was not altered by biotinylation. Moreover, consistent with the lack of biotin-CCL2 and biotin-CCL5 binding to HEK-huCCLR2 cells by flow cytometry, the binding affinities of label-free CCL2 and CCL5 to HEK-huCCRL2 cells could not be determined. While not all the ligands were tested in an unlabeled binding format, the corroborating results with chemerin, CCL2 and CCL5 between flow cytometry and SPRm binding assays provided support that our biotinylation assay preserved ligand activity. Using chemerin binding as a functional readout of CCRL2, we identified neutralizing antibodies (K097F7 and BZ5B8) for human and murine CCRL2, respectively.

CXCL8 induction of neutrophil activation and recruitment by binding CXCR2 and CXCR1 has been well-documented [39]. More recently, using CCRL2 genetically deficient mouse models and in vitro FRET experiments, CCRL2 has been proposed to promote neutrophil CXCL8 signaling by forming heterodimers with CXCR2 [17]. ACKR family members have also been shown to form heterodimers with signaling chemokine receptors to regulate cell activation and migration, such as with ACKR1/CCR5 and ACKR3/CXCR4, in the absence of ligand [40–42]. Here, we used pharmacological antibody tools to dissect the contribution of CCRL2 on CXCL8 signaling. Our identified CCRL2 neutralizing antibodies, K097F7 and BZ5B8, did not inhibit CXCL8-induced neutrophil degranulation in vitro or neutrophil peritoneal recruitment in vivo, respectively.

It is currently not known which residues are important for the CCRL2/CXCR2 interaction or whether chemerin (or other unidentified ligands) modify the formation and function of this heterodimer. Our data highlights differing neutrophil responses to CXCL8 when using CCRL2 pharmacological tools compared to that of previously reported CCRL2 deficient mice [17]. We cannot rule out that heterodimer functionality may have been preserved in our neutrophil studies given that the presence of CCRL2/CXCR2 heterodimers was not confirmed and assayed with our antibody tools. However, given that K097F7 and BZ5B8 neutralized chemerin binding and that CCRL2 deficient mice display attenuated neutrophil activation, our data suggest that the ligand binding function of CCRL2 is dispensable for regulation of a CXCL8 response in neutrophils. It is important to note that antibody molecular properties beyond chemerin neutralization were not assessed in this study. Therefore, it is possible that K097F7 or BZ5B8 may spatially hinder dimer formation at the cell surface. Alternatively, these antibodies could crosslink CCRL2 molecules, potentially enhancing aggregation of CCRL2/CXCR2 heterodimer. However, these potential antibody effects at the molecular level did not impact the downstream CXCL8-directed functions in neutrophil degranulation or migration.

Although CCRL2 neutralization did not modulate neutrophil migration in vivo, we observed a significant decrease in neutrophil CCRL2 surface expression following recruitment into the peritoneum. Previous work has demonstrated that CCRL2 undergoes ligand-independent internalization [38], however, our data suggest that CCRL2 is downregulated on neutrophils following activation with CXCL8. Given that CCRL2 is required for neutrophil integrin-mediated adhesion [17], it is tempting to speculate that CCRL2 promotes neutrophil recruitment and transmigration but internalizes once extravasation occurs to the site of inflammation. CCRL2 has also been reported to regulate CXCR2 expression given that bone marrow neutrophils deficient for CCRL2 express higher levels of CXCR2 [17]. Interestingly, neutrophil CXCR2 surface expression was not modulated in mice that received CXCL8 or CXCL8 following BZ5B8 dosing in our studies. This result also differs from CXCR2 regulation in human neutrophils as CXCL8 induces rapid CXCR2 internalization during neutrophil degranulation and migration processes [31]. Since mice do not express a direct CXCL8 homolog but can respond to human CXCL8 through activation of CXCR2 [1, 30, 43], CXCR2 may be differentially regulated by CXCL8 in mice. Alternatively, it has been proposed that CXCR2 initiates cellular changes needed for migration whereas CXCR1 directs chemotaxis to the site of inflammation [31, 44, 45]. While we did not monitor CXCR1, we cannot rule out the potential contribution of CXCR1 to direct neutrophil CXCL8 responses in vivo [46], which would occur independently of a CCRL2/CXCR2 axis. Further experimentation to understand the kinetics of the neutrophil CCRL2 response to CXCL8 would be needed to substantiate a temporal role for CCRL2 to regulate neutrophil migration.

CCRL2 genetically deficient mouse models have facilitated research on the function of CCRL2 in homeostasis and disease [16, 17, 47–49], however, studies demonstrating the functional translation to humans are still lacking. While little is known about the function of CCRL2 in monocytes, previous work showed that human CD14^+^ monocytes express CCRL2 [35, 50]. In humans, the majority of circulating monocytes comprise the classical subtype, which then differentiate into intermediate and non-classical monocytes when in circulation [51]. We also detected high expression of CCRL2 on human CD14^+^ monocytes and found that CCRL2 was expressed on classical, intermediate and non-classical monocytes, suggesting a role for CCRL2 throughout the monocyte lifecycle. Furthermore, characterization of CCRL2 expression on murine peritoneal leukocytes highlighted that CCRL2 expression was lower on monocytes than neutrophils and dendritic cells; a key difference from the high monocyte CCRL2 expression observed in our human whole blood profiling study. CCRL2 is also important for trafficking of activated, antigen loaded DC in mice [16] and CCRL2 expression is upregulated upon activation [16, 35]. However, CCRL2 expression was not previously detected at steady state on murine DCs [16]. Our profiling of CCRL2 on murine peritoneal DCs and human plasmacytoid and myeloid DCs provide support that CCRL2 is constitutively expressed on DCs, suggesting CCRL2 plays a role in DC homeostasis as well as response to inflammation. Except for two small subclusters, our finding that most B cells did not express CCRL2 corroborates previous reports [35, 36]. CCRL2 expression has been reported on pre-B cells and pro-B cells [34], however, these B cell subsets are bone marrow resident and would not be expected to be captured in our analysis of peripheral B cells. Thus, deeper immunophenotyping would be needed to characterize the B cell subsets that expressed CCRL2. Finally, we found that NKT cells are a novel cell type that expresses CCRL2. NKT cells have known roles for tumor defense by either directly killing target cells or secreting cytokines [52]. In murine models of lung tumor and melanoma, CCRL2 is protective against tumor progression by promoting NK cell activation and macrophage-T cell immunity, respectively [36, 47]. While a role for CCRL2 in NKT cell cancer immunity has not been reported, our findings raise questions on the potential diversity of CCRL2 in regulating cell activation.

In summary, we describe the utilization of live-cell flow cytometry and kinetic binding SPRm technologies to confirm chemerin as a direct ligand for CCRL2. Using these technologies, we identified human and mouse tool antibodies that neutralize the chemerin binding function of CCRL2. Unlike CCRL2 genetically deficient models, our identified CCRL2 pharmacological tool antibodies did not attenuate CXCL8-induced responses in human and murine neutrophils, suggesting that the ligand binding function of CCRL2 is dispensable for regulating a CXCL8 response. Additionally, we highlight a previously unappreciated regulatory role for CXCL8 to downregulate neutrophil CCRL2 following peritoneal transmigration, serving as a potential negative feedback loop to dampen CCRL2 cell surface function. Finally, given the limited information on CCRL2 function in human cells, we profiled CCRL2 expression on human peripheral blood leukocytes and found that CCRL2 is expressed on numerous cell types at steady state and identified NKT cells as a novel cell type that expresses CCRL2, prompting future studies to investigate CCRL2 cell type-specific functions.

## Supporting information

S1 Fig Assessment of biotin-labeled human ligands. SDS-PAGE assessment of biotin-labeled ligands under non-reducing (NR) and reducing (R) conditions shows expected molecular size and high purity for each ligand. Bands were visualized by Coomassie blue staining.
(TIF)

S2 Fig K_D_ determination of antibody binding to live cells using live-cell SPRm. (A) Schematic of SPRm instrument depicting the SPR and brightfield input, cell chamber and sample flow. (B) Representative SPRm data showing minimal binding of chemerin to parental HEK cells. (C) Representative SPRm data showing that clone K097F7 bound to HEK-huCCRL2 cells, (D) whereas clone K097F7 did not bind to parental HEK cells. (E) Representative SPRm data showing that clone 152254 bound to HEK-huCCRL2 cells, (F) whereas clone 152254 did not bind to parental HEK cells. (B-F) Data are representative of 3 experiments and squares overlaid on brightfield images indicate ROIs where binding events were detected by SPRm (*left)* and corresponding K_D_ histogram *(right)*. K_D_ values of 0 indicate that kinetics of binding could not be calculated due to minimal or no analyte binding.
(TIF)

S3 Fig Binding of label-free CCL2 and CCL5 to HEK-huCCRL2 cells. (A) Representative SPRm data showing minimal binding of CCL2 to HEK-huCCRL2 cells. (B) Representative SPRm data showing minimal binding of CCL5 to HEK-huCCRL2 cells. Data are representative of 3 experiments and squares overlaid on brightfield images indicate ROIs where binding events were detected by SPRm (*left)* and corresponding K_D_ histogram *(right)*. K_D_ values of 0 indicate that kinetics of binding could not be calculated due to minimal or no analyte binding.
(TIF)

S4 Fig Binding and ligand competition of additional anti-muCCRL2 antibodies. Flow cytometry detection of CCRL2 antibodies binding to HEK-muCCRL2 cells was performed with (A) clone 11n20 and (B) clone 498321, and binding is shown as MFI (Mean ± SEM, N = 3). (C) Unlabeled 11n20, 498321 or isotype control was incubated with HEK-muCCRL2 cells prior to addition of 10 nM biotin-labeled murine chemerin and antibody-ligand competition is shown as MFI (Mean ± SEM, N = 3).
(TIF)

S5 Fig Flow cytometry gating strategy to measure CCRL2 expression from whole blood human samples. (TIF)

S6 Fig Isolated UMAP analysis of CCRL2 expression in T cell, dendritic cell and monocyte populations. UMAP identified cell clusters for (A) CD4+ T cells, CD8+ T cells, and NKT cell populations, (B) myeloid and plasmacytoid dendritic cells and (C) classical, non-classical and intermediate monocytes were depicted *(left)*. Relative CCRL2 PE intensity levels were overlaid onto the same clustered populations of the UMAP (*right)*.
(TIF)

S1 Table CCRL2 antibodies used for flow cytometry binding assay. (DOCX)

S2 Table Flow cytometry antibodies used in murine peritoneum leukocyte profiling in CXCL8 neutrophil recruitment model. (DOCX)

S3 Table Flow cytometry antibodies used in whole blood leukocyte profiling and isolated neutrophil expression analysis. (DOCX)

S1 Raw images (PDF)
