# Supplementary figures and images for: Studies with neutralizing antibodies suggest CXCL8-mediated neutrophil activation is independent of C-C motif chemokine receptor-like 2 (CCRL2) ligand binding function
Source: PLoS One. 2023 Jan 20;18(1):e0280590. doi: 10.1371/journal.pone.0280590 (PMC9858354; doi:10.1371/journal.pone.0280590)

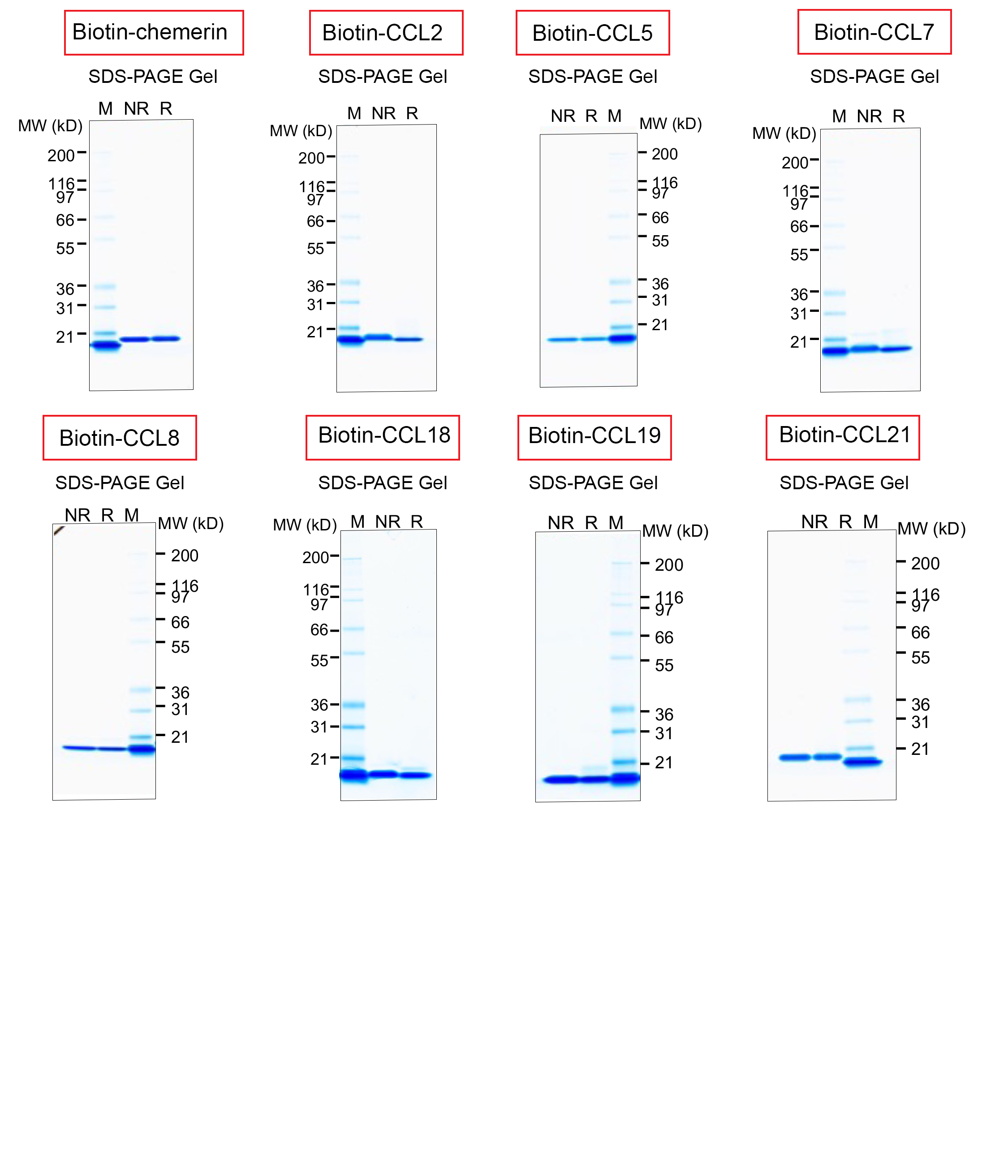

Supplement: S1 Fig — SDS-PAGE assessment of biotin-labeled ligands under non-reducing (NR) and reducing (R) conditions shows expected molecular size and high purity for each ligand. Bands were visualized by Coomassie blue staining. (TIF) [file pone.0280590.s001.tif]

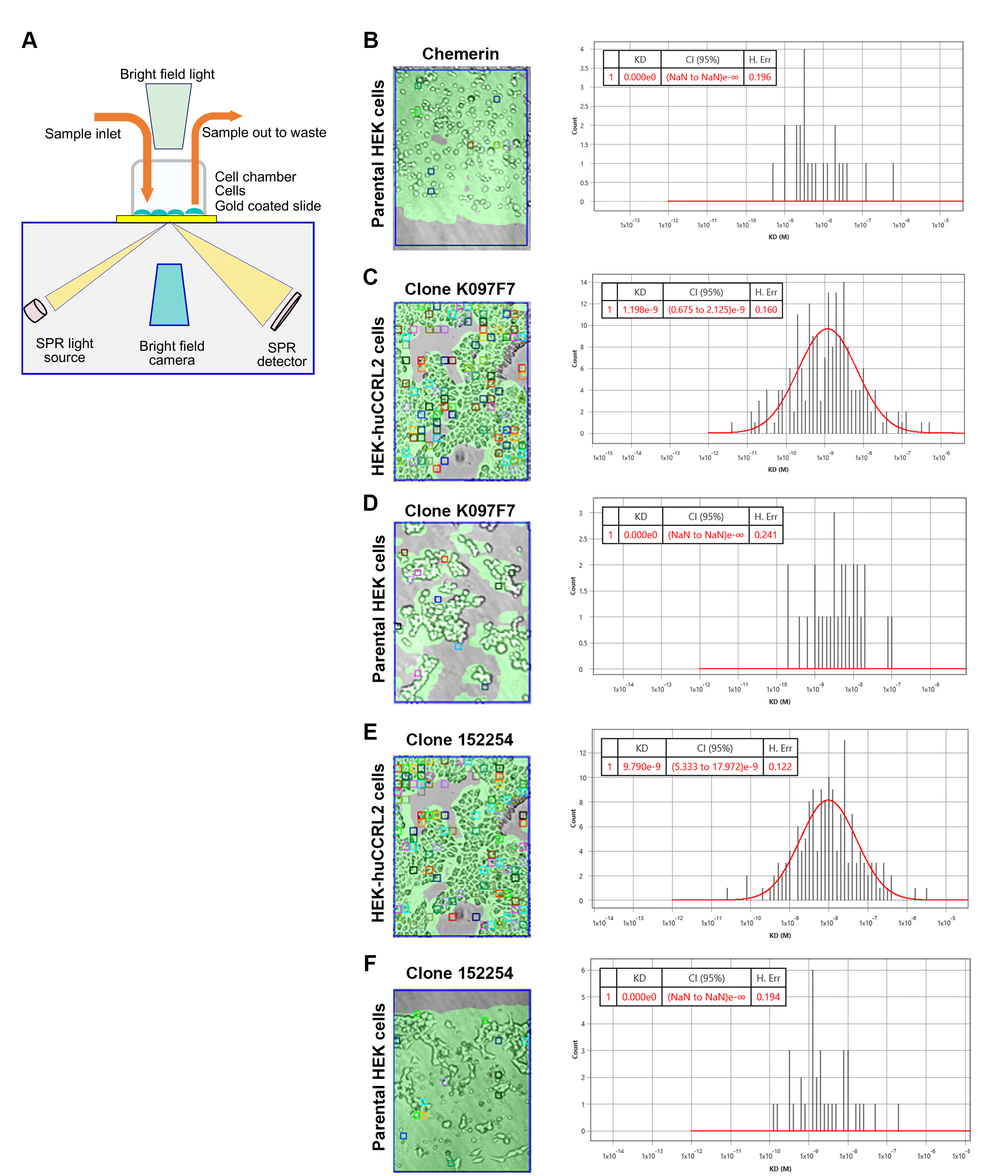

Supplement: S2 Fig — (A) Schematic of SPRm instrument depicting the SPR and brightfield input, cell chamber and sample flow. (B) Representative SPRm data showing minimal binding of chemerin to parental HEK cells. (C) Representative SPRm data showing that clone K097F7 bound to HEK-huCCRL2 cells, (D) whereas clone K097F7 did not bind to parental HEK cells. (E) Representative SPRm data showing that clone 152254 bound to HEK-huCCRL2 cells, (F) whereas clone 152254 did not bind to parental HEK cells. (B-F) Data are representative of 3 experiments and squares overlaid on brightfield images indicate ROIs where binding events were detected by SPRm (left) and corresponding KD histogram (right). KD values of 0 indicate that kinetics of binding could not be calculated due to minimal or no analyte binding. (TIF) [file pone.0280590.s002.tif]

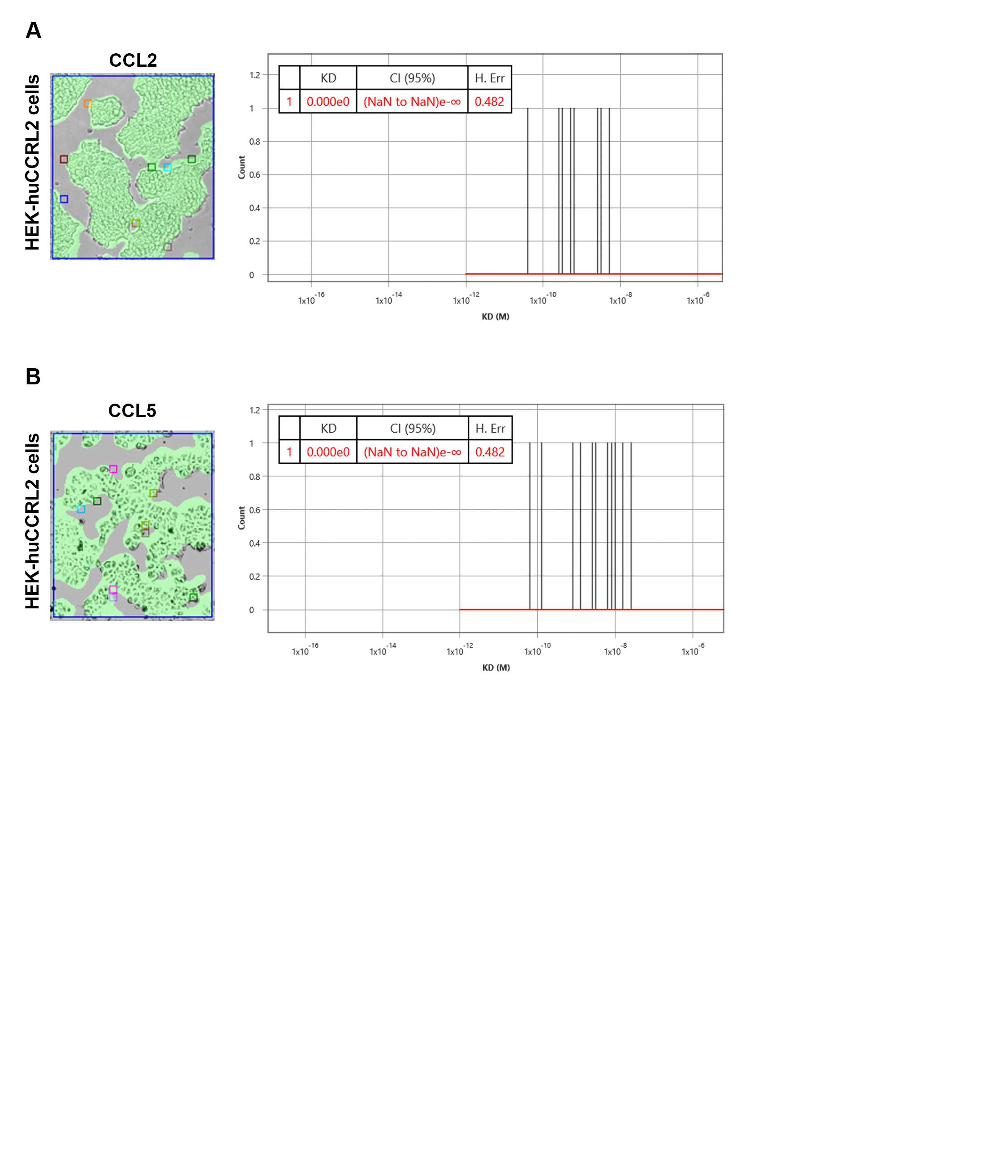

Supplement: S3 Fig — (A) Representative SPRm data showing minimal binding of CCL2 to HEK-huCCRL2 cells. (B) Representative SPRm data showing minimal binding of CCL5 to HEK-huCCRL2 cells. Data are representative of 3 experiments and squares overlaid on brightfield images indicate ROIs where binding events were detected by SPRm (left) and corresponding KD histogram (right). KD values of 0 indicate that kinetics of binding could not be calculated due to minimal or no analyte binding. (TIF) [file pone.0280590.s003.tif]

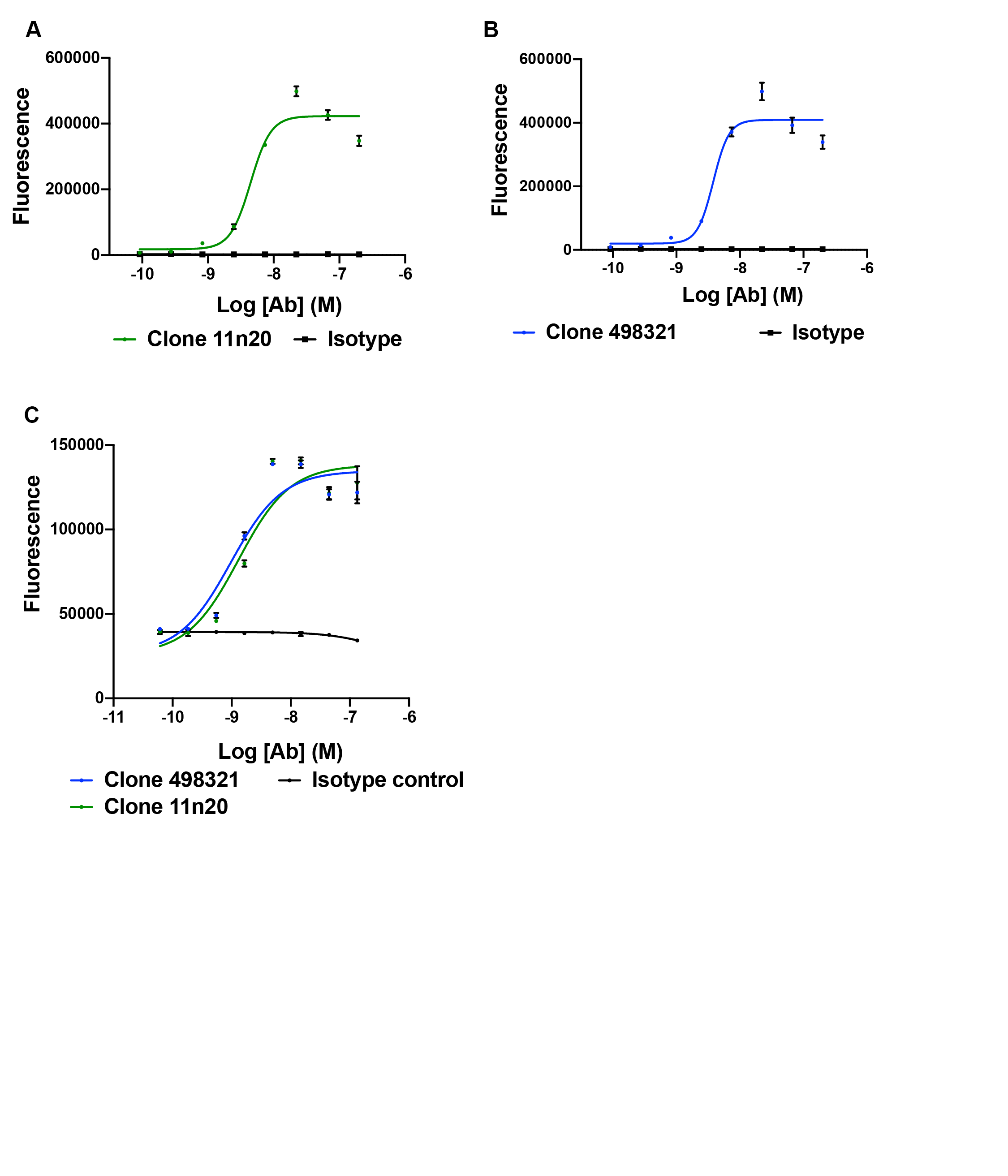

Supplement: S4 Fig — Flow cytometry detection of CCRL2 antibodies binding to HEK-muCCRL2 cells was performed with (A) clone 11n20 and (B) clone 498321, and binding is shown as MFI (Mean ± SEM, N = 3). (C) Unlabeled 11n20, 498321 or isotype control was incubated with HEK-muCCRL2 cells prior to addition of 10 nM biotin-labeled murine chemerin and antibody-ligand competition is shown as MFI (Mean ± SEM, N = 3). (TIF) [file pone.0280590.s004.tif]

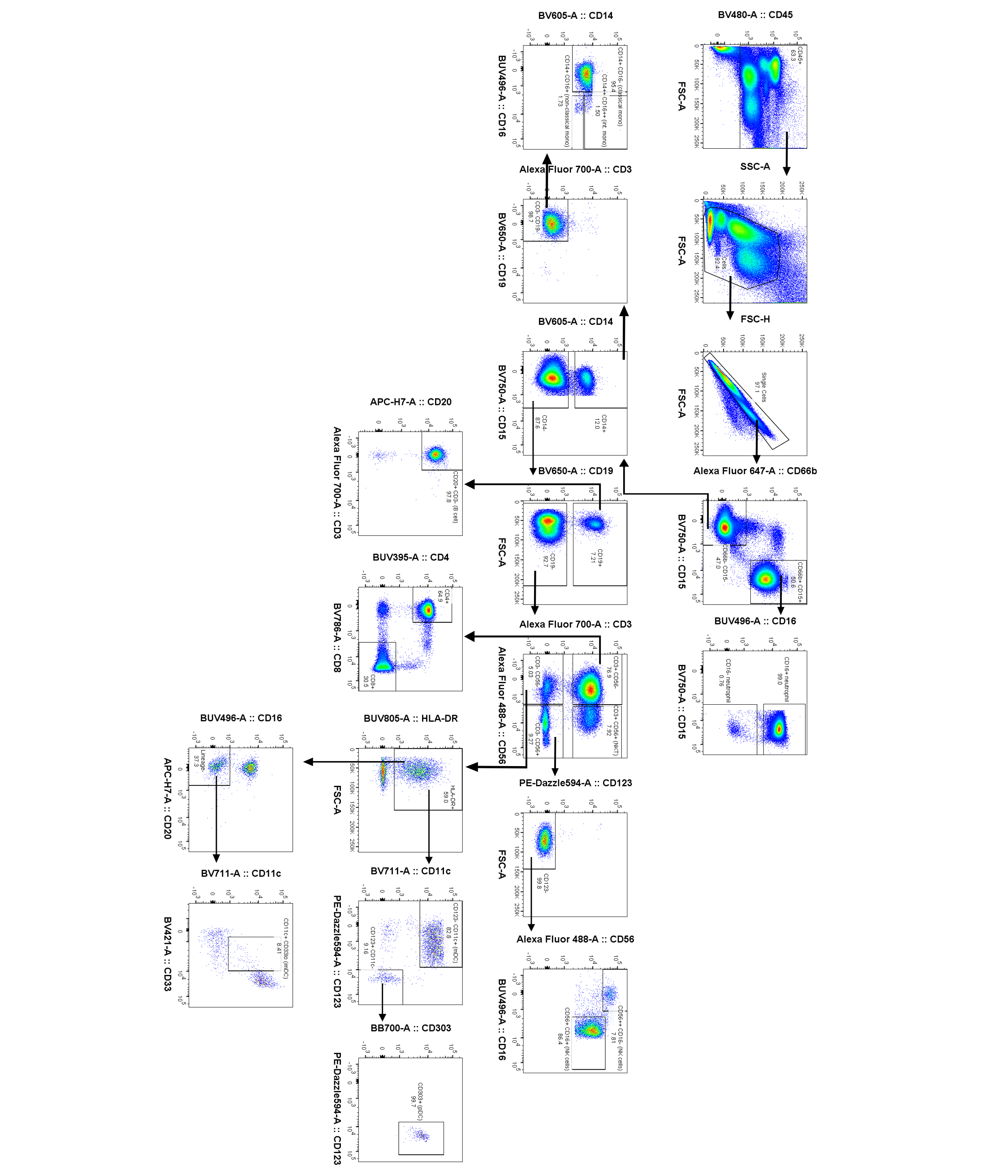

Supplement: S5 Fig — (TIF) [file pone.0280590.s005.tif]

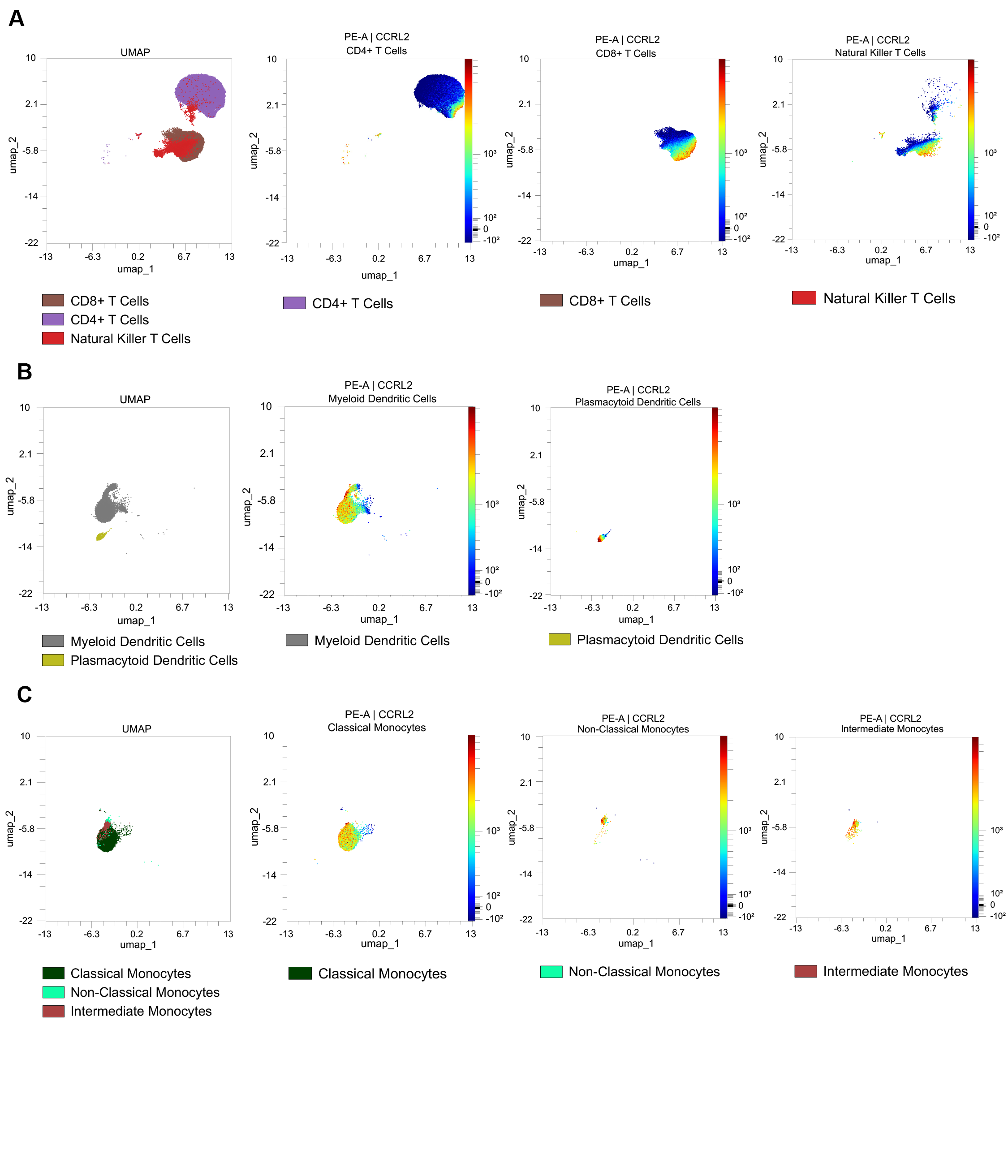

Supplement: S6 Fig — UMAP identified cell clusters for (A) CD4+ T cells, CD8+ T cells, and NKT cell populations, (B) myeloid and plasmacytoid dendritic cells and (C) classical, non-classical and intermediate monocytes were depicted (left). Relative CCRL2 PE intensity levels were overlaid onto the same clustered populations of the UMAP (right). (TIF) [file pone.0280590.s006.tif]
