# Supplementary material for: Studies with neutralizing antibodies suggest CXCL8-mediated neutrophil activation is independent of C-C motif chemokine receptor-like 2 (CCRL2) ligand binding function
Source: PLoS One. 2023 Jan 20;18(1):e0280590. doi: 10.1371/journal.pone.0280590 (PMC9858354; doi:10.1371/journal.pone.0280590)
Supplement: S1 Table — (DOCX) [file pone.0280590.s007.docx]

## S1 Table. CCRL2 antibodies used for flow cytometry binding assay.

| **Species** | **Host** | **Clone** | **Vendor** |
| --- | --- | --- | --- |
| Human | Mouse IgG2a | K097F7 | Biolegend |
| Human | Mouse IgG2b | 152211 | R&D Systems |
| Human | Mouse IgG2b | 152254 | R&D Systems |
| Human | Mouse IgG2a | 5A15 | MyBioSource |
| Mouse | Rat IgG2a | BZ5B8 | Millipore |
| Mouse | Rat IgG1 | 498321 | R&D Systems |
| Mouse | Rat IgG1 | 11n20 | Lifespan Biosciences |
