# Supplementary material for: Studies with neutralizing antibodies suggest CXCL8-mediated neutrophil activation is independent of C-C motif chemokine receptor-like 2 (CCRL2) ligand binding function
Source: PLoS One. 2023 Jan 20;18(1):e0280590. doi: 10.1371/journal.pone.0280590 (PMC9858354; doi:10.1371/journal.pone.0280590)
Supplement: S2 Table — (DOCX) [file pone.0280590.s008.docx]

## S2 Table. Flow cytometry antibodies used in murine peritoneum leukocyte profiling in CXCL8 neutrophil recruitment model.

| **Specificity** | **Fluorochrome** | **Clone** | **Vendor** |
| --- | --- | --- | --- |
| CD16/CD32 (Fc block) |  | 2.4G2 | BD Biosciences |
| CD45 | Alexa Fluor 700 | 30-F11 | BD Biosciences |
| CD11b | BUV395 | M1/70 | BD Biosciences |
| CD49b | BV421 | HMα2 | BD Biosciences |
| CD11c | BV605 | HL3 | BD Biosciences |
| CD45R/B220 | PerCPCy5.5 | RA3-6B2 | BD Biosciences |
| Ly6G | FITC | 1A8 | BD Biosciences |
| CD182 (CXCR2) | Alexa Fluor 647 | SA045E1 | Biolegend |
| CCRL2 | PE | BZ2E3 | BD |
| 7-AAD Viability Staining Solution |  |  | ThermoFisher |
| IgG2a, k Isotype | PE | MOPC-173 | Biolegend |
| IgG1, k Isotype | PE | MOPC-21 | BD Biosciences |
| CD123 | PE-Dazzle 594 | 6H6 | Biolegend |
| CD66b | Alexa Fluor 647 | G10F5 | Biolegend |
| CD3 | Alexa Fluor 700 | UCHT1 | Biolegend |
| CD20 | APC-H7 | 2H7 | BD |
| LIVE/DEAD | Near-IR |  | Invitrogen |
