# Supplementary material for: Studies with neutralizing antibodies suggest CXCL8-mediated neutrophil activation is independent of C-C motif chemokine receptor-like 2 (CCRL2) ligand binding function
Source: PLoS One. 2023 Jan 20;18(1):e0280590. doi: 10.1371/journal.pone.0280590 (PMC9858354; doi:10.1371/journal.pone.0280590)
Supplement: S3 Table — (DOCX) [file pone.0280590.s009.docx]

## S3 Table. Flow cytometry antibodies used in whole blood leukocyte profiling and isolated neutrophil expression analysis.

| **Specificity** | **Fluorochrome** | **Clone** | **Vendor** |
| --- | --- | --- | --- |
| CD4 | BUV395 | SK3 | BD Biosciences |
| CD16 | BUV496 | 3G8 | BD Biosciences |
| CD11b | BUV737 | ICRF44 | BD Biosciences |
| HLA-DR | BUV805 | G46-6 | BD Biosciences |
| CD33 | BV421 | WM53 | BD Biosciences |
| CD45 | BV480 | HI30 | BD Biosciences |
| CD45 | Alexa Fluor 488 | HI30 | Biolegend |
| CD14 | BV605 | M5E2 | BD Biosciences |
| CD19 | BV650 | SJ25C1 | BD Biosciences |
| CD11c | BV711 | Bly6 | BD Biosciences |
| CD15 | BV750 | W6D3 | BD Biosciences |
| CD15 | BV421 | W6D3 | BD Biosciences |
| CD8 | BV786 | G42-8 | BD Biosciences |
| CD56 | Alexa Fluor 488 | NCAM 16.2 | BD Biosciences |
| CD303 | BB700 | V24-785 | BD Biosciences |
| CCRL2 | PE | K097F7 | Biolegend |
| CXCR2 | PE | 6C6 | BD Biosciences |
| IgG2a, k Isotype | PE | MOPC-173 | Biolegend |
| IgG1, k Isotype | PE | MOPC-21 | BD Biosciences |
| CD123 | PE-Dazzle 594 | 6H6 | Biolegend |
| CD66b | Alexa Fluor 647 | G10F5 | Biolegend |
| CD3 | Alexa Fluor 700 | UCHT1 | Biolegend |
| CD20 | APC-H7 | 2H7 | BD |
| LIVE/DEAD | Near-IR |  | Invitrogen |
