# Supplementary material for: Studies with neutralizing antibodies suggest CXCL8-mediated neutrophil activation is independent of C-C motif chemokine receptor-like 2 (CCRL2) ligand binding function
Source: PLoS One. 2023 Jan 20;18(1):e0280590. doi: 10.1371/journal.pone.0280590 (PMC9858354; doi:10.1371/journal.pone.0280590)

Biotin-chemerin

X X M NR R X X X

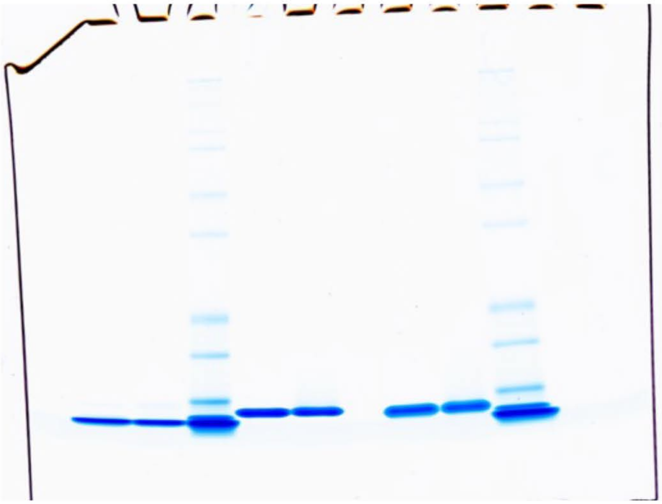

Biotin-CCL2

X X M NR R X X X X X

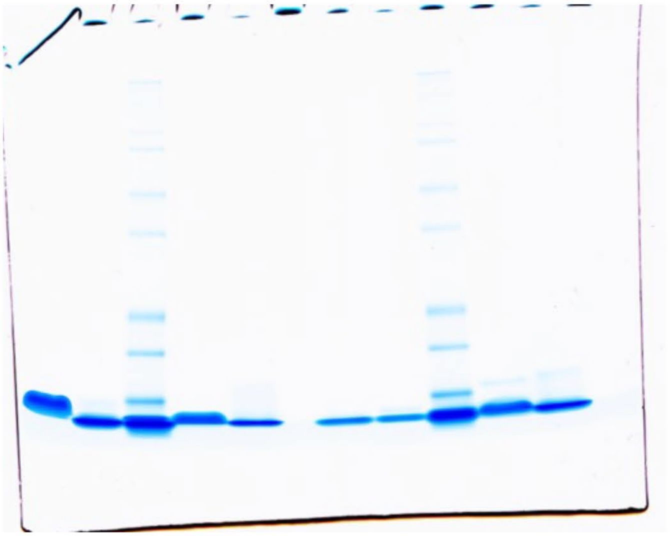

Biotin-CCL5

X X X X X NR R M X X

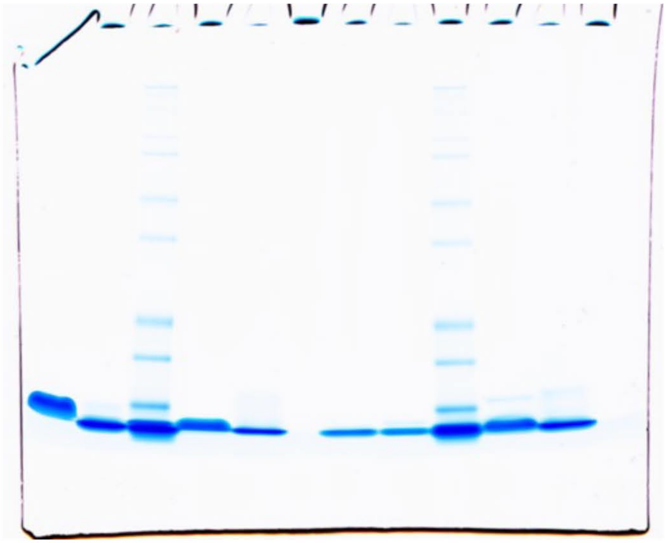

Biotin-CCL7

X X X X X X X M NR R

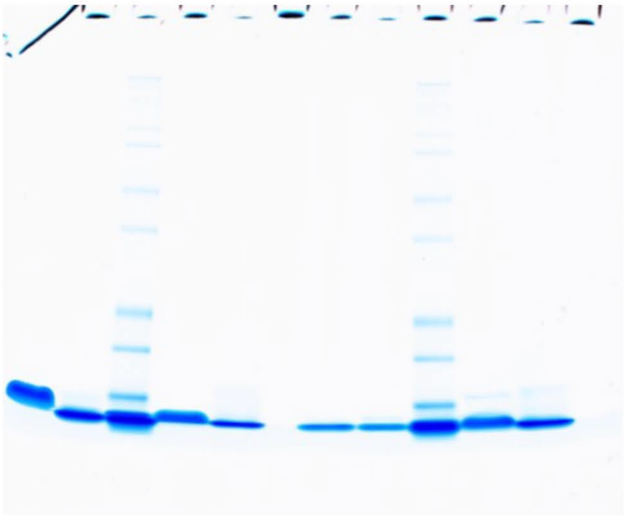

Biotin-CCL8

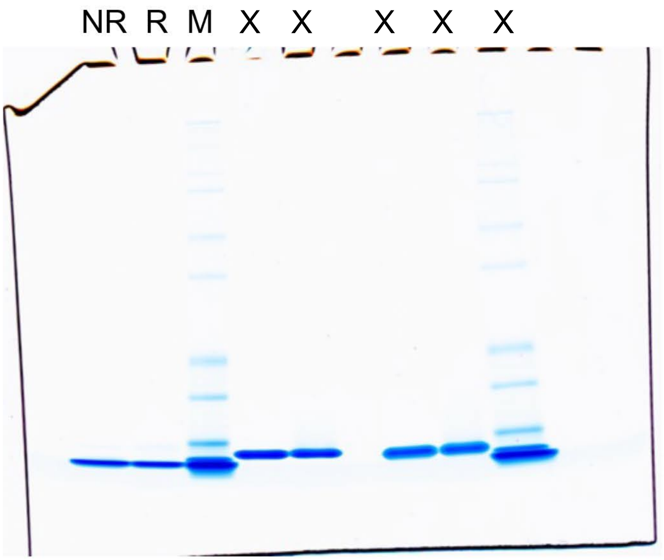

Biotin-CCL18

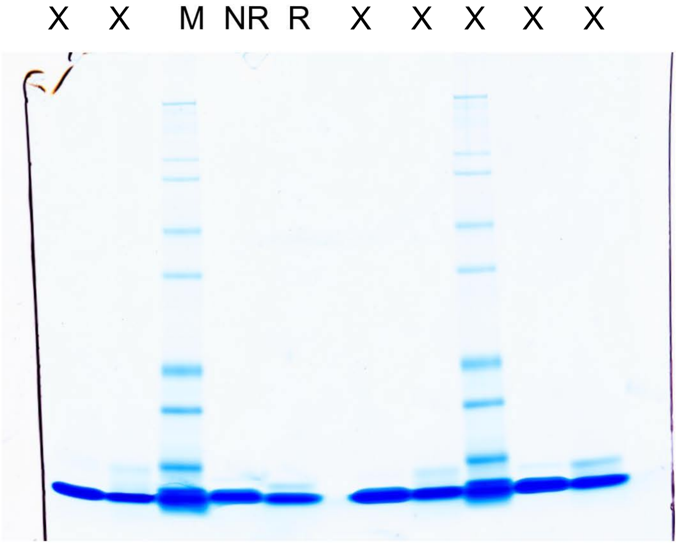

Biotin-CCL19

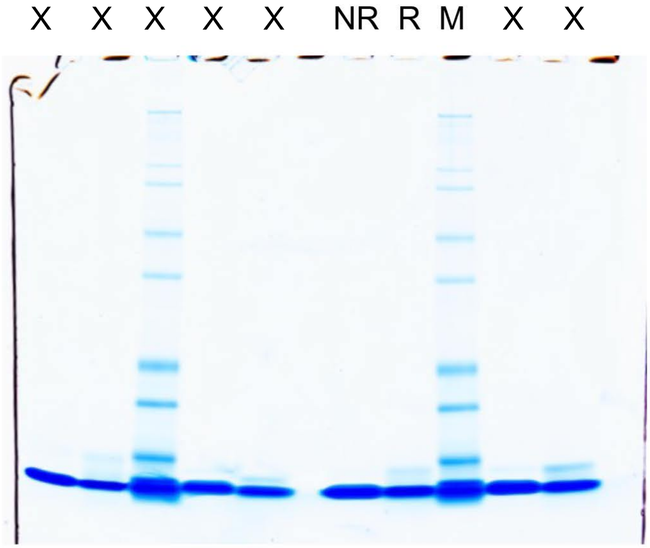

Biotin-CCL21

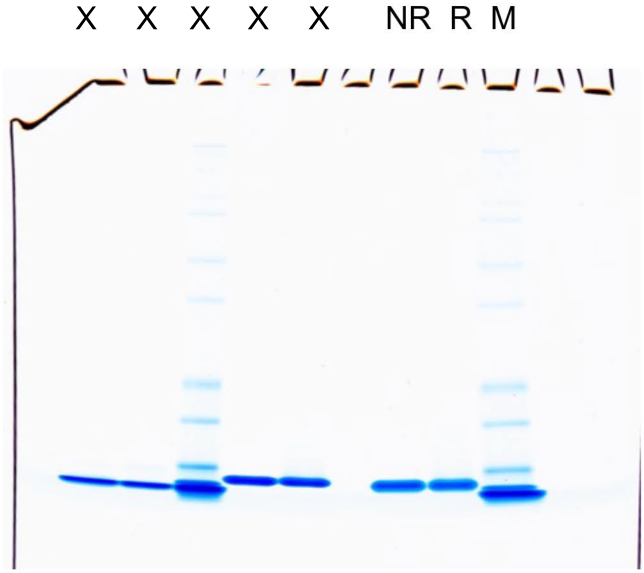

Supplement: S1 Raw images — (PDF) [file pone.0280590.s010.pdf]
